# Determinants of male healthcare utilization in Switzerland: does gender identity and migration background matter?

**DOI:** 10.1186/s12913-026-14185-y

**Published:** 2026-02-27

**Authors:** Marietta Lieb, Christopher Zaiser, Nora M. Laskowski, Roland Müller, Markus Theunert, Georgios Paslakis

**Affiliations:** 1 Medical Faculty, Ruhr-University Bochum, University Clinic for Psychosomatic Medicine and Psychotherapy, Campus East-Westphalia, Virchowstr. 65, 32312 Luebbecke, Germany; 2 Männer.ch, Competence Centre for Men’s Health, Bern, Switzerland

**Keywords:** Swiss health survey, Male health care utilization, Gender identity, Transgender, Non-binary, Migration background, Minorities

## Abstract

**Background:**

Men often exhibit lower rates of healthcare utilization. Particularly vulnerable groups—such as men belonging to gender minority groups and men with a migration background—may face additional barriers in accessing care. This study examined healthcare utilization patterns across diverse male gender subgroups in Switzerland, with attention to the role of migration status, while accounting for key sociodemographic factors.

**Methods:**

We analyzed a subset of the Swiss Health Survey (SHS) 2022, a nationally representative dataset of the general Swiss population. Our sample included individuals falling into one of the three groups: cisgender men, transgender men and gender diverse assigned male at birth (AMAB) individuals (assigned male at birth and identifying as nonbinary or another gender). Healthcare utilization was assessed through the number of general practitioner (GP) and specialist physician (SP) visits, use of mental health services, and utilization of complementary medicine. Additionally, we evaluated perceived quality of care for GP and SP visits. Four regression models were conducted to examine associations between healthcare utilization, gender identity, migration background and sociodemographic characteristics.

**Results:**

After weighting, our study comprised 3,505,801 male cases, representing an unweighted sample size of *N* = 8,699. Among gender subgroups, transgender men showed higher utilization across all types of health care services, including GP and SP visits and mental health care compared to cisgender men. Gender diverse AMAB individuals reported lower use of GP and SP services, as well as complementary medicine, but higher use of mental health services. Perceived quality of GP and SP care was more often moderate or poor among gender diverse AMAB individuals. First-generation migrants used GP services slightly more frequently but accessed other services less often than men without migration background. Second-generation migrants showed similar patterns to those without migration background, except for lower use of complementary medicine. Key sociodemographic variables were associated with notable differences in health care utilization.

**Discussion:**

Healthcare utilization in Switzerland differs by male gender identity and migration background. These findings underscore the need for more inclusive, identity-sensitive healthcare with a focus on gender diverse AMAB individuals. Addressing structural and access-related barriers is essential to ensure equitable healthcare utilization among male migrant populations, especially first-generation migrants.

**Supplementary Information:**

The online version contains supplementary material available at 10.1186/s12913-026-14185-y.

## Background

Men’s health has received comparatively little attention in public health research, even though they represent a particularly vulnerable group in many respects: Globally, men have a lower life expectancy [1–4], show higher rates of certain chronic conditions (e.g., cardiovascular disease, diabetes) [2], as well as higher incidence of unintentional injuries [2] and higher physical occupational hazards [3, 5] compared to women. They further exhibit elevated suicide rates [2, 3, 6] and tend to engage more frequently in risky behaviors, including smoking, alcohol and drug consumption, as well as poorer dietary habits [3, 7, 8]. Alongside their heightened risk profile and greater susceptibility to certain negative health conditions, studies show that men are less likely to recognize disease symptoms [9] and are less inclined to utilize preventative health services, screening and primary health care compared to women, ultimately contributing to poorer health outcomes [3, 10, 11]. Especially regarding the utilization of mental health services, there is a considerable gender gap. Men are less likely to seek mental health care [12] and less inclined to acknowledge the potential benefits of psychotherapy [13]. A Swiss study supports these findings, suggesting that men consistently use healthcare services less frequently than women [14]. This underutilization of healthcare is not only limited to conventional evidence-based services but is also observed for complementary medicine (e.g., acupuncture, homeopathy, osteopathy, shiatsu etc.), which men tend to use less frequently than women [15, 16]. Although the clinical effectiveness of complementary medicine remains controversial and evidence is limited [17–20], it has steadily gained popularity in Switzerland, with approximately 28.9% of the population reporting use of such therapies [15, 16].

Adverse health-related behaviors in men are often attributed to sociocultural factors, such as traditional masculinity norms [12]: Utilization of (mental) health care may be misperceived as a sign of weakness and vulnerability, contradicting the still prevailing societal ideal of a “self-sufficient and strong” man, thereby leading to stigmatization [11, 12, 21]. This is further exacerbated by structural barriers that may prevent men from seeking help, including a shortage of male practitioners and predominantly feminine-oriented care environments [11, 12].

While these findings may apply to men in general, differences in healthcare utilization are likely to emerge when adopting a broader, more gender-diverse perspective. Previous studies demonstrated that gender minorities such as transgender (men) and nonbinary individuals were less likely to regularly see or have access to a personal health care provider [22, 23], were more likely to be non-adherent to medication or forgo necessary treatments [23] and receive fewer routine check-ups [22, 24] compared to cisgender individuals. These patterns are often attributed to fear of discrimination and fear of poor treatment, as well as a lack of adequately trained professionals, resulting in reduced, delayed, or avoided health care utilization, ultimately contributing to poorer physical and mental health outcomes [23, 25–29]. This is especially worrying given that transgender (men) and nonbinary individuals consistently report worse physical and mental health than their cisgender counterparts [22, 30–33]. Such disparities are often explained through stress-based frameworks. The Gender Minority Stress Model (GMSM) [34] is an extension of the original Minority Stress Model (MSM) [35], which explains how distal stressors (e.g. stigma and discrimination) and proximal stressors (e.g. internalized stigma) affect health outcomes in the lesbian, gay, and bisexual (LGB) population. The GMSM extends the MSM by specifically addressing gender-specific stressors, such as stressful events including non-affirmation (being addressed with wrong pronouns), harassment, violence and/ or discrimination due to gender identity or gender expression and proximal stressors, such as internalized transphobia and expectations of gender-based violence. These stressors, according to the GMSM contribute to higher risk for adverse mental and physical health outcomes among gender minorities [34]. Studies on the utilization of healthcare services among transgender men and nonbinary individuals in Switzerland are limited. However, one Swiss study combining data from the Swiss Health Survey (SHS 2012 & 2017) and a study on LGBT (lesbian, gay, bisexual and transgender) health [29] reported that transgender men and women (*N* = 279) and nonbinary individuals (*N* = 243) had higher odds of consulting a psychologist compared to gay or bisexual cisgender individuals (*N* = 1542). No significant differences were observed in consultations with general practitioners (GPs) when controlling for age, gender, nationality, and education. These findings align with evidence of the greater mental health challenges commonly reported by transgender and nonbinary individuals [32]. In Switzerland, psychotherapy or a psychiatric evaluation is also often part of gender-affirming care, which is generally covered by health insurance. This may partly contribute to the higher utilization of mental health services observed in this population. However, the same Swiss study also found that 16.2% of LGBT individuals forgo healthcare due to a lack of trust in medical professionals or institutions—more than twice the 6.9% observed in the general Swiss population—highlighting persistent barriers to access and potential experiences of discrimination. Moreover, less than two thirds of transgender and gender diverse people reported satisfaction with the mental health services they received [26]. Perceived quality of care has been found to be a factor influencing healthcare utilization and may further shape how transgender and gender diverse people may engage with these services [36].

Another vulnerable group of men frequently marginalized within healthcare systems consists of individuals with a migration background. Despite often facing poorer health outcomes than the native population [37], they make comparatively less use of available healthcare services: A German review [38] highlights lower healthcare utilization among individuals with a migration background, particularly in areas such as specialist care, medication use, therapist consultations, counselling, disease prevention and complementary medicine. In contrast, no disparities were observed in visits to GPs. The review identified first-generation and second-generation individuals with a two-sided migration background as groups with particularly low levels of healthcare utilization. Similarly, another Swiss study [39], drawing on data from the SHS 2012, found that individuals with a migration background tend to visit doctors less frequently than those without. This difference was primarily attributed to individuals with first-generation migration background and those from culturally diverse backgrounds (i.e., from countries whose typical societal value patterns differ from those prevalent among Swiss non-migrants), who were significantly less likely to seek medical care. However, no notable differences were observed between individuals with a second-generation migration background and those without.

Besides gender identity and migration background, there are also other sociodemographic factors that have been found to play a role in healthcare utilization, such as age [15, 36], education [14, 15, 36], marital status [14, 36], employment status [36, 40], household structure [41, 42], sexual orientation [29], residential environment [36], and language region [14]. There is some evidence suggesting that older age, higher education, being married, being unemployed, living alone, heterosexual orientation and living in remote areas are associated with higher healthcare use [14, 15, 29, 36, 40–42]. However, findings are not consistent across studies and may vary depending on the type of healthcare considered.

Overall, these findings highlight persistent inconsistencies in research on healthcare utilization among vulnerable groups such as men belonging to gender minority groups and men with a migration background. Moreover, there is a lack of research differentiating between subgroups of gender minorities, with insufficient studies focusing specifically on men. It also remains unclear to what extent other sociodemographic variables might influence this relationship and how perceived quality of care may differ across these groups and shape their healthcare-seeking behavior. To address these gaps, the present study aims to examine how male gender identities and migration background contribute to healthcare utilization in Switzerland, while accounting for key sociodemographic factors. By identifying potential risk factors and specific vulnerable groups, this research seeks to inform the development of targeted interventions and health policies to improve access to appropriate healthcare services for gender-diverse and migrant men.

## Methods

### Sampling and procedures

This study is based on data from the 2022 SHS conducted by the Swiss Federal Statistical Office [43]. The SHS is a large-scale, nationally representative survey administered every five years. It captures a broad range of information related to individual health status, behavior, and determinants, and has incorporated core components of the European Health Interview Survey (EHIS) since 2007 to allow cross-national comparisons.

The 2022 survey targeted all individuals aged 15 years or older residing in private households across Switzerland, including foreign nationals with valid residence or short-term permits of at least one year. A stratified random sampling framework was applied, using cantonal and municipal population registers that are updated quarterly. Cantons served as strata, with minimum sample sizes allocated to each major region to ensure regional representativeness. While the core national sample comprised 10,000 individuals, 17 cantons and the city of Zurich opted for sample expansions to enable more detailed cantonal-level analyses.

Data collection followed a mixed-mode survey design. The majority of participants completed computer-assisted telephone interviews (CATI), while computer-assisted personal interviews (CAPI) were offered as alternatives. Each interview lasted approximately 30 min. Following the interviews, participants were invited to complete a self-administered written questionnaire either online (74%) or via paper-and-pencil (26%). In total, 21,930 interviews were completed (response rate: 36.2%), and 19,137 valid written questionnaires were returned (response rate: 90.1%).

The survey was conducted continuously throughout the year 2022, with quarterly sampling evenly distributed to account for potential seasonal variation in health behaviors and conditions. Interviews were conducted in the three official languages of Switzerland—German, French, and Italian—corresponding to the linguistic regions. To ensure high data quality and reliability, rigorous quality control procedures were implemented throughout the data collection and processing stages. These included real-time plausibility checks during interviews, manual validation of inconsistencies in the written questionnaires, and cross-verification of demographic data with official population registers. All data were thoroughly cleaned and anonymized in compliance with Swiss data protection legislation. Participation in the survey was voluntary, and the confidentiality of all respondents was strictly protected.

Only participants who completed both the interview and the written survey were included in the analyses; the respective datasets were merged prior to analysis. Additionally, the data used in this study are owned by the Swiss Federal Statistical Office and cannot be shared publicly due to licensing restrictions. Further details on SHS data collection procedures are available in the SHS methods overview [43].

### Measures

In order to examine the utilization of diverse health care services among Swiss men, we used a subset of data derived from the SHS 2022 focusing on (1) GP and specialist physician (SP) health care, (2) mental health care utilization and (3) use of complementary medicine.

In order to assess utilization of general and specialist health care, the number of visits to GPs and SPs during the past 12 months were assessed, respectively. Perceived quality of care was evaluated for both GP and SP visits using a 5-point Likert scale, ranging from excellent (1), very good (2), good (3), moderate (4), to bad (5). Mental health care utilization was assessed via self-report, using a binary response format (no = 0/yes = 1), to indicate whether participants had received treatment for a mental health problem within a 12 month period. The type of service utilized for mental health treatment was assessed, categorized as follows: Psychologist/Psychotherapist (no = 0/yes = 1), Psychiatrist (no = 0/yes = 1), GP (no = 0/yes = 1), Other Physician (no = 0/yes = 1), Complementary and alternative medicine practitioner (no = 0/yes = 1), Other non-medical therapist (no = 0/yes = 1). Due to its rising popularity in Switzerland [15, 16], we further assessed the utilization of complementary medicine by combining the number of visits to an osteopath, alternative practitioner, and the use of other complementary treatments (e.g., acupuncture, homeopathy, shiatsu, classical massage etc.) in the past 12 months into a single binary variable (no = 0/yes = 1). In addition, we assessed if the person had supplementary insurance for complementary medicine (no = 0, yes = 1). In Switzerland, certain complementary medicine methods are covered by the mandatory basic health insurance if performed by certified physicians [44]. However, treatments by non-medical practitioners typically require fee-based supplemental or optional insurance, or must be paid out-of-pocket by the patient [15].

The questions used to assess health care utilization can be found in Supplementary File 1.

### Sample definition

For the categorization of gender, the SHS employs a two-step approach, consistent with established survey methodology recommendations [45, 46]. Two self-report items were administered: the first assessed sex assigned at birth in a binary format (male/female), and the second captured participants’ current gender identity with several response options (man, woman, non-binary, other, I don’t know). Based on the combination of sex assigned at birth and current gender identity, we categorized participants accordingly. For our analyses, we included only individuals who fell into one of the following three groups: (a) cisgender men—those assigned male at birth who currently identify as men; (b) transgender men—those assigned female at birth who currently identify as men; and (c) gender diverse assigned male at birth (AMAB) individuals —those assigned male at birth and who currently identify as nonbinary or with an unspecified gender identity. We excluded individuals assigned male at birth who currently identify as women (transgender women), as well as those assigned male at birth but did not report their current gender identity.

For individuals with migration background, we distinguished three categories: (a) individuals without migration background, defined as persons born in Switzerland with at least one parent also born in Switzerland, (b) first-generation migrants, defined as persons born abroad who themselves migrated to Switzerland, with both parents also born abroad, and (c) second-generation (or higher) migrants, defined as persons born in Switzerland whose parents were both born abroad and migrated to Switzerland.

Other sociodemographic variables were categorized as follows: Marital status was categorized into two groups: (a) married – includes registered partnerships and (b) not married – includes those who were single, widowed, divorced, unmarried or had a dissolved registered partnership. Employment status had also two groups: (a) employed and (b) not employed, with the latter including non-employable men (unemployed and currently not available for the job market, such as pensioners, invalids, houseman/housewife, in training) and unemployed men (unemployed and available for the job market within the next two weeks). Sexual orientation included the following two categories: (a) heterosexual orientation and (b) non-heterosexual orientation, with the latter including bisexual, homosexual and other sexual orientations. For the number of persons living in the household, we dichotomized the data into (a) 1 Person (living alone) and (b) > 1 Persons (living with others). For age, we used three categories: (a) 15-39years, (b) 40–64 years and (c) 65 + years, a categorization suggested by the SHS data management. For education we also had three groups: (a) compulsory education – 9 years including primary school, (b) secondary education – includes vocational training and general education, and (c) tertiary education – includes universities and higher vocational training. Residential areas were categorized into three types: (a) urban, (b) intermediate – such as dense peri-urban and rural zones and (c) rural. Finally, we accounted for the three Swiss language areas (a) German-speaking, (b) French-speaking and (c) Italian-speaking in our sample.

### Weighting

The 2022 SHS [43] provides a representative sample of the Swiss population. To ensure that the survey results accurately reflect the demographic structure of the Swiss population and allow for valid generalizations, the data were weighted using the survey weights provided by the Swiss Health Survey, which adjust for the sampling design, non-response, and key demographic characteristics. The weighting process involved comparing the sample composition with official population benchmarks and adjusting for discrepancies in factors such as region of residence, gender, age, nationality, marital status, and household size. Base weights accounted for the stratified sampling design and selection probabilities, as well as initial adjustments for survey non-participation. To further reduce bias from differential non-response, model-based adjustments were applied using response behavior patterns. Finally, a calibration step aligned the sample with known population distributions to ensure representativeness. All analyses in this study were conducted using the weighted data, which enhances the validity and generalizability of the findings. We reported unweighted and weighted sample sizes for all descriptive data and statistical analyses.

### Statistical analysis

All data were processed and analyzed using IBM SPSS Statistics© (Version 30) [47] and Python (Version 3.13.5) [48] with NumPy version 2.3.0 [49] and SciPy version [50] 1.16.1 packages. Figures were created with Microsoft Excel 2016© [51]. Descriptive statistics included frequencies, means (Ms), medians (MDs), standard deviations (SDs), and ranges. For the pre-analysis, we examined differences in health care utilization and perceived quality of care among male gender identities and groups differing in migration status. Due to uneven data distribution, we applied Kruskal-Wallis tests to assess group differences for continuous variables. For categorical variables, group comparisons were performed using Chi-square tests. We depicted effect sizes accordingly (ε² for Kruskal-Wallis and Cramér’s V for Chi²-tests). We further conducted several post-hoc tests to examine specific group differences. Following significant Kruskal-Wallis tests, we conducted pairwise Mann-Whitney U-tests to explore group differences. Additionally, we calculated Cliff’s Delta (δ) [52] as a post-hoc effect size measure, given its suitability for non-parametric, ordinal, and skewed data. Following significant Chi-square tests, we performed pairwise comparisons using separate Chi-square tests with Bonferroni correction. To quantify the magnitude of group differences, we calculated Cramér’s V as an effect size measure.

For the main analysis, we conducted separate negative binomial regressions for the number of GP and SP visits. Negative binomial regression was chosen because the outcome variables are count data that exhibited overdispersion, meaning the variance exceeds the mean, which violates the assumptions of Poisson regression [53]. For each negative binomial regression, we reported regression coefficients (Bs), standard errors (SEs), incidence rate ratios (IRRs = Exp[B]), Wald statistics, p-values, and corresponding 95% confidence intervals (CIs). IRRs are calculated by exponentiating the regression coefficients (B) from the negative binomial model. An IRR greater than 1 indicates a higher, and an IRR less than 1 indicates a lower expected count of the outcome per unit increase in the predictor. IRRs were used to evaluate the magnitude of the relationships between predictors and outcomes. Model fit was assessed using a likelihood ratio test, comparing the full model with predictors to the intercept-only model. A significant test indicated that the inclusion of predictors improved model fit. We further conducted separate binary logistic regression analyses for utilization of complementary medicine and treatment due to mental health problems. Binary logistic regression was chosen because both outcomes are binary, allowing us to estimate the probability of each event based on the predictors. Reported statistics included Bs, SEs, odds ratios (Exp[B]), Wald statistics, p-values, and corresponding 95% CIs. Odds ratios (ORs) were used to interpret and compare the strength of associations between predictors and outcomes. Model fit was assessed using the likelihood ratio test, and the explained variance was evaluated with Nagelkerke’s R². Given the well-documented influence of sociodemographic factors on healthcare utilization [14, 29, 36, 40–42], we controlled for age, education, marital status, employment status, household structure, sexual orientation, residential environment, and language region in all regression models. Multicollinearity was assessed for all predictors prior to the analyses, and no issues were detected. To further test the robustness of our models and potential mediator effects, we included the respective outcome variables as predictors in the other models. This was done only for variables that showed significant correlations, assuming that different health care utilization services (GP and SP visits) are related. These correlations were assessed using Spearman’s correlation. Improvement in model fit was assessed by comparing the two models, using deviance, Akaike Information Criterion (AIC), Bayesian Information Criterion (BIC), log-likelihood, and Pearson chi-square per degree of freedom (df). For the utilization of complementary medicine, we conducted a separate robustness analysis using binary logistic regression to examine the potential effect of having supplemental health insurance coverage for complementary treatments, while also testing the robustness of the model and potential mediator effects. Improvement in model fit was assessed by comparing the two models using − 2 Log-Likelihood and measures of explained variance (Cox & Snell R² and Nagelkerke R²). All statistical analyses were based on complete cases, with a significance threshold of *p* < .05 applied to all tests.

## Results

### Sample characteristics

Analyses were performed using survey weights, resulting in a weighted sample of *N* = 3,505,801 men encompassing cisgender men, transgender men, and gender diverse AMAB individuals. The corresponding unweighted analytic sample comprised *N* = 8,699 men. Descriptive details for all variables are presented in Table 1.

**Table 1:** Sample characteristics

**Variable**		
**Age**	**Mean (SD), median (range)**	
	**Weighted**	**Unweighted**
	48.07 (18.85); 48 (15-100)	52.46 (18.94); 54 (15-100)
	**N (%)** ^¥^	
	**Weighted**	**Unweighted**
**Male gender**		
Cisgender men	3,841,962 (99.3)	8,648 (99.4)
Transgender men	17,448 (0.5)	36 (0.4)
Gender diverse AMAB individuals	6,392 (0.2)	16 (0.2)
Missing	0 (0)	0 (0)
**Migration status**		
Without	2,168,988 (62.4)	5,964 (68.9)
First-generation	1,013,762 (29.1)	2,086 (24.1)
≥ Second-generation	295,589 (8.5)	602 (7.0)
Missing	27,462 (0.8)	47 (0.5)
**Age categories**		
15-39 years	1,290,996 (36.8)	2,267 (26.1)
40-64 years	1,473,808 (42.0)	3,930 (45.2)
65+ years	740,996 (21.1)	2,502 (28.8)
Missing	0 (0)	0 (0)
**Sexual orientation**		
Heterosexual	3,094,890 (92.8)	7,720 (93.8)
Non-heterosexual	239,368 (6.8)	508 (6.2)
Missing	171,542 (4.9)	471 (5.4)
**Marital status**		
Not married	1,732,374 (49.4)	5,144 (59.1)
Married	1,773,426 (50.6)	3,555 (40.9)
Missing	0 (0)	0 (0)
**Persons in household**		
1 Person	2,858,432 (81.6)	7,362 (84.7)
>1 Person	644,659 (18.4)	1,330 (15.3)
Missing	0 (0)	0 (0)
**Education**		
Compulsory	449,615 (12.9)	8,568 (9.9)
Secondary	1,466,905 (42.1)	3,616 (41.7)
Tertiary	1,568,959 (45.0)	4,193 (48.4)
Missing	20,320 (0.6)	34 (0.4)
**Employment status**		
Not employed	1,007,608 (28.7)	5911 (68.0)
Employed	2,497,806 (71.3)	2786 (32.0)
Missing	387 (0.01)	2 (0.02)
**Residential area**		
Urban	2,160,333 (61.6)	5,213 (59.9)
Intermediate	782,623 (22.3)	1,888 (21.7)
Rural	562,844 (16.1)	1598 (18.4)
Missing	0 (0)	0 (0)
**Language areas**		
German-speaking	2,534,392 (72.3)	6,158 (70.8)
French-speaking	829,246 (23.7)	1,991 (22.9)
Italian-speaking	142,163 (4.1)	550 (6.3)
Missing	0 (0)	0 (0)

Note: SD=standard deviation; ^¥^Percentages are calculated based on non-missing cases. Missing values are shown separately

### Utilization of health care and perceived quality of care in men

Table 2 displays the overall utilization of health care services in the total male sample.

**Table 2:** Utilization of health care services

**GP visits**	**Mean (SD), Median (range)**	
	**Weighted**	**Unweighted**
Total	2.74 (3.13), 2 (0-52)	2,76 (3.17), 2 (0-52)
	**N (%)** ^¥^	
No (no visits at all)	217,398 (8.2)	517 (7.7)
Yes (at least one visit)	2,422,191 (91.8)	6,217 (92.3)
Missing	866,212 (24.7)	1,965 (22.6)
**Quality of last GP visit **	**N (%)** ^¥^	
Excellent	815,883 (33.8)	2,056 (33.1)
Very good	1,058,369 (43.8)	2,790 (45.0)
Good	445,187 (18.4)	1,118 (18.0)
Moderate	78,939 (3.3)	205 (3.3)
Bad	17,182 (0.7)	36 (0.6)
Missing	1,090,243 (31.1)	2,494 (28.7)
**SP visits **	**Mean (SD), Median (range)**	
Total	1.58 (4.39), 0, 0-99	1.58 (4.18), 0, 0-99
	**N (%)** ^¥^	
No (no visits at all)	1,935,192 (55.4)	4,491 (51.8)
Yes (at least one visit)	1,560,263 (44.6)	4,183 (48.2)
Missing	10,346 (0.3)	25 (0.3)
**Quality of Last SP visit**	**N (%)** ^¥^	
Excellent	570,230 (36.6)	1,540 (36.9)
Very good	615,632 (39.6)	1,730 (41.5)
Good	291,108 (18.7)	728 (17.5)
Moderate	60,896 (3.9)	135 (3.2)
Bad	18,287 (1.2)	38 (0.9)
Missing	1,949,647 (55.6)	4,528 (52.1)
**Treatment due to mental health problems**	**N (%)** ^¥^	
Missing	2,160 (0.1)	5 (0.1)
No	3,291,799 (94.0)	8,246 (94.8)
Yes	211,842 (6.0)	448 (5.2)
Mental health service utilized*		
Psychologist/Psychotherapist	94,585 (2.7)	203 (2.3)
Psychiatrist	113762 (3.2)	241 (2.8)
GP	37,452 (1.1)	78 (0.9)
Other physician	6,859 (0.2)	16 (0.2)
Complementary/Alternative medicine practitioner	7,342 (0.2)	22 (0.3)
Other non-medical therapist	1,677 (0.05)	6 (0.1)
**Utilization of complementary medicine**	**N (%)** ^¥^	
No	2,683,325 (76.9)	6,591 (76.2)
Yes	806,071 (23.1)	2,063 (23.8)
Missing	16,405 (0.5)	45 (0.5)
**Supplemental health insurance for complementary medicine**		
Yes	1,593,462 (55.6)	4,319 (59.2)
No	1,270,222 (44.4)	2,975 (40.8)
Missing	642,117 (18.3)	1,405 (16.2)

Note: All visits and treatment utilizations refer to a 12 months period; *multiple services may have been used simultaneously during the 12 months period. GP = General Practitioner, SP = Specialist Physician; ¥Percentages are calculated based on non-missing cases. Missing values are shown separately

### Group differences in health care utilization

Table 3 presents the differences in healthcare utilization between groups. All Kruskal-Wallis and Chi-square tests across male gender groups and migration status were statistically significant (*p* < .001, effect sizes negligible to small). Bonferroni-corrected post-hoc tests confirmed significant pairwise differences (*p*<.05) with varying effect sizes.

The Cliff’s Delta effect sizes for pairwise comparisons of GP visits by male gender were as follows: gender diverse AMAB vs. cisgender showed a small (δ = 0.15), gender diverse AMAB vs. transgender demonstrated small to moderate effect (δ = 0.22), and cisgender vs. transgender revealed a negligible effect (δ = − 0.08). For SP visits, all male group differences were negligible to small (Cliff’s δ: transgender vs. cisgender = –0.11, transgender vs. gender diverse AMAB = –0.05, cisgender vs. gender diverse AMAB = –0.09). Pairwise Chi²-tests revealed significant differences across male gender identities in psychological treatment, complementary medicine use and supplemental health insurance for complementary medicine (all *p* < .001, Bonferroni-adjusted), with negligible to small effect sizes (Cramér’s V = 0.011–0.116 for psychological treatment; 0.005–0.07 for complementary medicine; 0.009-0.074 for supplemental health insurance for complementary medicine).

Cliff’s Delta effect sizes for pairwise comparisons of GP visits by migration status were δ = –0.05 (negligible) for no migration background vs. second-generation, δ = –0.09 (negligible) for no migration background vs. first-generation, and δ = 0.07 (negligible) for the comparison between the two migration groups. The estimated Cliff’s Delta effect sizes for pairwise comparisons of SP visits by migration status were δ = − 0.04 (negligible) for first-generation versus second-generation, δ = 0.11 (negligible to small) for first-generation versus no migration background, and δ = 0.09 (negligible) for second-generation vs. no migration background. Pairwise Chi²-tests revealed statistically significant group differences in psychological treatment, complementary medicine use and supplemental health insurance for complementary medicine by migration status (all *p* < .001, Bonferroni-adjusted). However, effect sizes were negligible for both psychological treatment (Cramér’s V = 0.02–0.05) and complementary medicine (Cramér’s V = 0.03–0.07) and negligible to small for supplemental health insurance for complementary medicine (Cramer’s V= 0.04-0.113).

**Table 3:** Group differences in health care utilization

**Male gender**			**Cisgender men**	**Transgender men**	**Gender diverse AMAB individuals**	**Analytical Sample Size (** ***N*** **)**	Kruskal-Wallis Test/ Chi²-Test*
			Mean (SD), Median				
GP visits			2.76 (3.31), 2	3.32 (3.35), 2	1.92 (1.25), 2	6,734 (U) 2,639,572 (W)	H(2) = 415.05, ε²=0.00016
SP visits			1.58 (4.40), 0	1.86 (2.80), 1	1.40 (2.51), 1	8,674 (U) 3,495,458 (W)	H(2) = 1026.82, ε²=0.00029
			N (%)				
Treatment MHP		Yes No	207,111 (6.0) 3,272,690 (94.0)	3,951 (22.6) 13,498 (77.4)	780 (12.2) 5,612 (87.8)	8,694 (U) 3,503,642 (W)	χ²(2) = 8942.04, Cramér’s V=0.05
Complementary medicine		Yes No	798,149 (23.0) 2,667,407 (77.0)	6,146 (35.2) 11,303 (64.8)	1,777 (27.8) 4,615 (72.2)	8,654 (U) 3,489,397 (W)	χ²(2) = 1532.32, Cramér’s V=0.02
SHI for complementary medicine		Yes No	1,586,367 (55.7) 1,259,576 (44.3)	4,620 (37.6%) 7,673 (62.4%)	2,476 (45.4) 2,973 (54.6)	7,294 (U) 2,863,685 (W)	χ²(2) = 1865.62, Cramér’s V=0.03
**Migration status**			**Without MB**	**First-generation**	**Second-generation**		**Kruskal-Wallis Test/ Chi²-Test**
			Mean (SD), Median				
GP visits			2.69 (3.11), 2	2.89 (3.38), 2	2.62 (2.44), 2	6,702 (U) 2,624,063 (W)	H(2) = 2469.19, ε²=0.00094
SP visits			1.64 (4.54), 0	1.48 (4.14), 0	1.54 (4.25), 0	8,627 (U) 3,467,996 (W)	H(2) = 6934.75, ε²=0.002
			N (%)				
Treatment MHP	Yes No		132,365 (6.1) 2,035,325 (93.9)	54,257 (5.4) 958,643 (94.6)	23,835 (8.1) 271,753 (91.9)	8,647 (U) 3,476,178 (W)	χ²(2) = 2975.32, Cramér’s V=0.03
Complementary medicine	Yes No		544,733 (25.2) 1,614,697 (74.8)	192,284 (19.1) 814,631 (80.9)	64,712 (21.9) 230,876 (78.1)	8,907 (U) 3,467,933 (W)	χ²(2) = 14788.99, Cramér’s V=0.07
SHI for complementary medicine	Yes No		1,078,013 (59.6) 730,137 (40.4)	390,783 (47.6) 431,051 (52.4)	115,809 (53.3) 101,314 (46.7)	7,263 (U) 2,847,107 (W)	χ²(2) = 33,862.53, Cramér’s V=0.11

Note: SD= standard deviation, GP= general practitioner, SP= specialist physician, treatment MHP= treatment due to mental health problems, SHI=Supplemental Health Insurance. MB=migration background, *all results were significant on a *p*<.001 level. W=Weighted, U=Unweighted

### Group differences in perceived quality of care

We further examined the quality of care for GP and SP visits across male gender identities and migration status. Results are displayed in Table 4. Chi-square tests indicated statistically significant differences in perceived quality of care across male gender groups and migration status (*p* < .001). Effect sizes ranged from negligible to small. Bonferroni-adjusted post-hoc analyses confirmed significant pairwise differences (*p* < .001), though the magnitude of effects varied between comparisons.

For GP visit quality, effect sizes for post-hoc tests were negligible between cisgender and transgender (Cramér’s V = 0.03) and between cisgender and gender diverse AMAB (Cramér’s V = 0.07), but large between transgender and gender diverse AMAB (Cramér’s V = 0.58). For SP visit quality, the effect size between cisgender and transgender individuals was negligible (Cramér’s V = 0.05), small between cisgender and gender diverse AMAB (Cramér’s V = 0.12), and large between transgender and gender diverse AMAB (Cramér’s V = 0.58).

Between groups of different migration status, the effect sizes for GP visit quality were consistently negligible (Cramér’s V = 0.02-0.05). The same applies to SP visit quality where effect sizes were generally negligible (Cramér’s V = 0.04-0.05), except for the difference between first- and second-generation migration background, which showed a small effect (Cramér’s V = 0.10).

**Table 4:** Group differences in perceived quality of care

**Male gender**	**Cisgender men**	**Transgender men**	**Gender diverse AMAB individuals**	**Analytical Sample Size (** ***N*** **)**	Chi²-Test*
Quality of last GP visit					
Excellent	810,026 (33.8)	3,070 (24.4)	2,787 (57.5)	6,205 (U) 2,415,559 (W)	χ²(8)=14,379.541, Cramér’s V=0.06
Very good	1,053,087 (43.9)	5,018 (39.9)	264 (5.5)		
Good	440,800 (18.4)	4,052 (32.2)	335 (6.9)		
Moderate	77,503 (3.2)	444 (3.5)	991 (20.5)		
Bad	16,715 (0.7)	0 (0)	467 (9.6)		
Quality of last SP visit					
Excellent	568,034 (36.8)	1,267 (14.3)	929 (24.0)	4,171 (U) 1,556,155 (W)	χ²(8)=25,699.104, Cramér’s V=0.09
Very good	611,321 (39.6)	3,529 (39.7)	782 (20.2)		
Good	287,510 (18.6)	3,460 (39.0)	138 (3.6)		
Moderate	59,365 (3.8)	360 (4.1)	1,172 (30.3)		
Bad	17,169 (1.1)	266 (3.0)	853 (22.0)		
**Migration status**	**Without MB**	**First-generation**	**Second-generation**		**Chi²-Test**
Quality of last GP visit					
Excellent	504,541 (33.0)	239,998 (35.3)	66,625 (33.9)	6,178 (U) 2,403,459 (W)	χ²(8)=6412,863, Cramér’s V=0.04
Very good	696,064 (45.6)	272,688 (40.1)	83,121 (42.3)		
Good	268,017 (17.5)	137,437 (20.2)	39,301 (20.0)		
Moderate	48,577 (3.2)	23,626 (3.5)	6,518 (3.3)		
Bad	10,369 (0.7)	5,496 (0.8)	1,081 (0.5)		
Quality of last SP visit					
Excellent	365,280 (36.1)	152,834 (37.2)	48,793 (38.8)	4,153 (U) 1,547,088 (W)	χ²(8)=6164,699, Cramér’s V=0.05
Very good	406,149 (40.2)	153,141 (37.3)	53,367 (42.4)		
Good	187,332 (18.5)	85,724 (20.9)	15,873 (12.6)		
Moderate	39,386 (3.9)	16,148 (3.9)	5,363 (4.3)		
Bad	12,352 (1.2)	2,910 (0.7)	2,436 (1.9)		

Note: GP= general practitioner, SP= specialist physician, MB=migration background; *all results were significant on a *p*<.001 level. W=Weighted, U=Unweighted

### Regression models predicting health care utilization in men

#### General practitioner visits

**Fig. 1:**
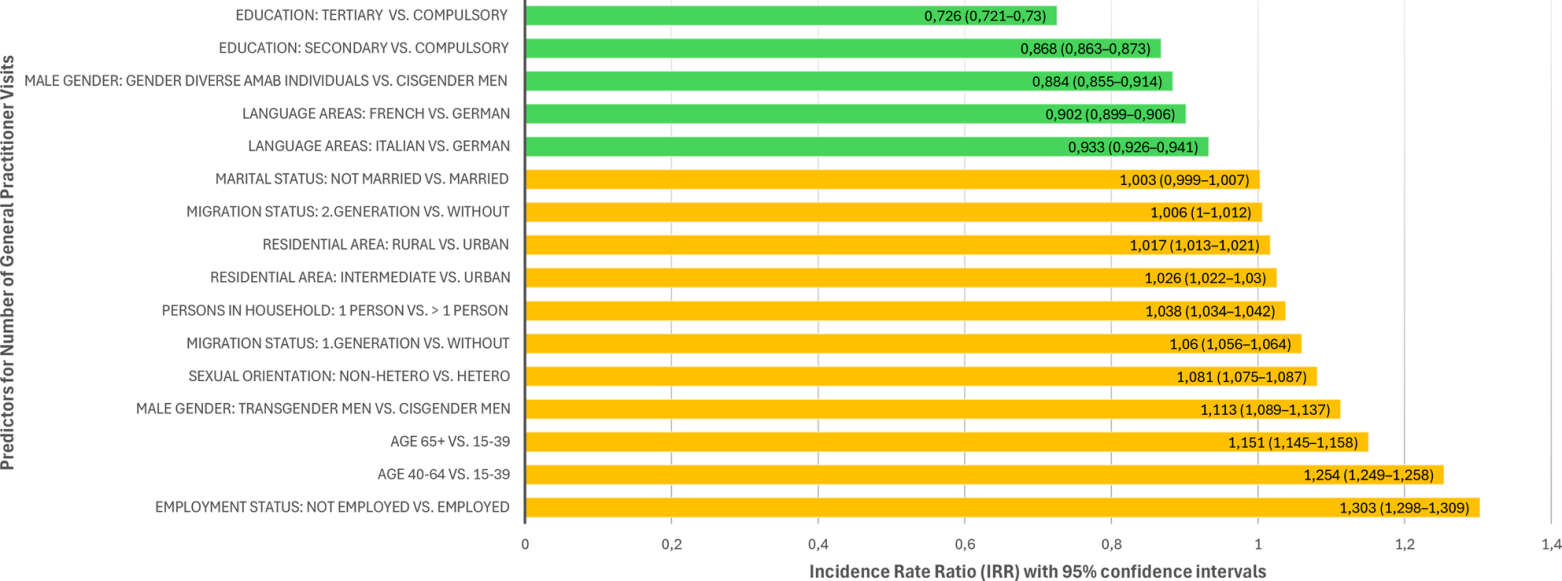
Incidence rate ratios of predictors for number of general practitioner visits

A negative binomial regression was conducted to examine predictors of the number of visits to a GP in the past 12 months. Model fit was assessed using the likelihood ratio test, which indicated that the full model with predictors significantly improved the fit compared to the intercept-only model, χ² (16) = 902,900, *p*<.001. All predictors were statistically significant except for marital status (not being married vs. being married, *p*=.105). The strongest predictors for a higher number of GP visits were unemployment (IRR = 1.303), older age (40–64 and 65+, IRR = 1.254 and IRR = 1.151) and being a transgender man (IRR = 1.113). Other risk factors with lower IRRs were non-heterosexual orientation (IRR = 1.081), first-generation migration background (IRR of 1.060), living in a one-person household (IRR = 1.038) and residential area (intermediate: IRR = 1.026; rural: IRR = 1.017). Although the statistical evidence was weaker compared to other predictors, the association found for second-generation migration background was still statistically significant (OR = 1.006, *p* = .041). In contrast, higher education levels (secondary and tertiary school education, IRR = 0.868 and 0.726), being a gender diverse AMAB individual (IRR = 0.884) and living in a French- (IRR=0.902) and Italian-speaking region (IRR=0.933) were associated with fewer visits. All predictors and IRRs are displayed in the supplementary Table 1, a graphical display is depicted in Fig. 1.

#### Specialist physician visits

**Fig. 2:**
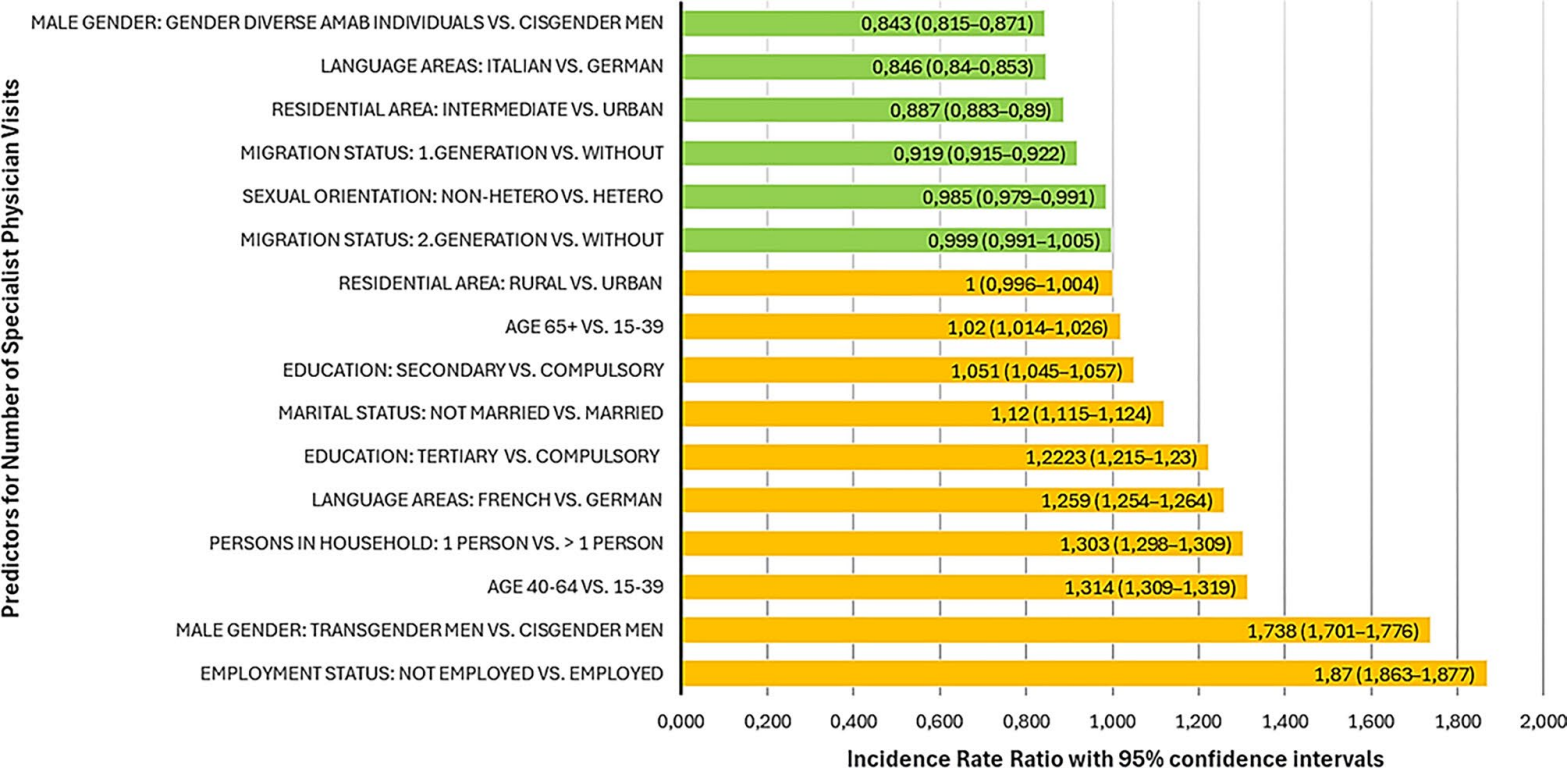
Incidence rate ratios of predictors for number of specialist physician visits

For the negative binomial regression to examine the predictors for the number of SP visits in the past 12 months, the full model with predictors significantly improved model fit compared to the intercept-only model, χ²(3,878,660) = 14,320,426, *p* < .001 and is depicted in supplementary Table 2. All predictors were statistically significant (*p*<.001), except for living in a rural area and having a migration background (second-generation). The predictors for a higher number of SP visits, in descending order, were unemployment (IRR = 1.870), being a transgender man (IRR = 1.738), being middle aged (40-64years, IRR = 1.314), living in a one-person household (IRR = 1.303), living in a French-speaking region (IRR = 1.259), tertiary education (IRR = 1.223), not being married (IRR = 1.120), secondary education (IRR = 1.051) and age 65+ (IRR = 1.020). The most important factors associated with lower visits were being a gender diverse AMAB individual (IRR=0.843) and residing in an Italian-speaking (IRR=0.846) and in an intermediate area (IRR=0.887). First-generation migration background had an IRR of 0.919 and non-heterosexual orientation an IRR of 0.985. A graphical display of all predictors and their respective IRRs is depicted in Fig. 2.

#### Utilization of treatment due to mental health problems

**Fig. 3:**
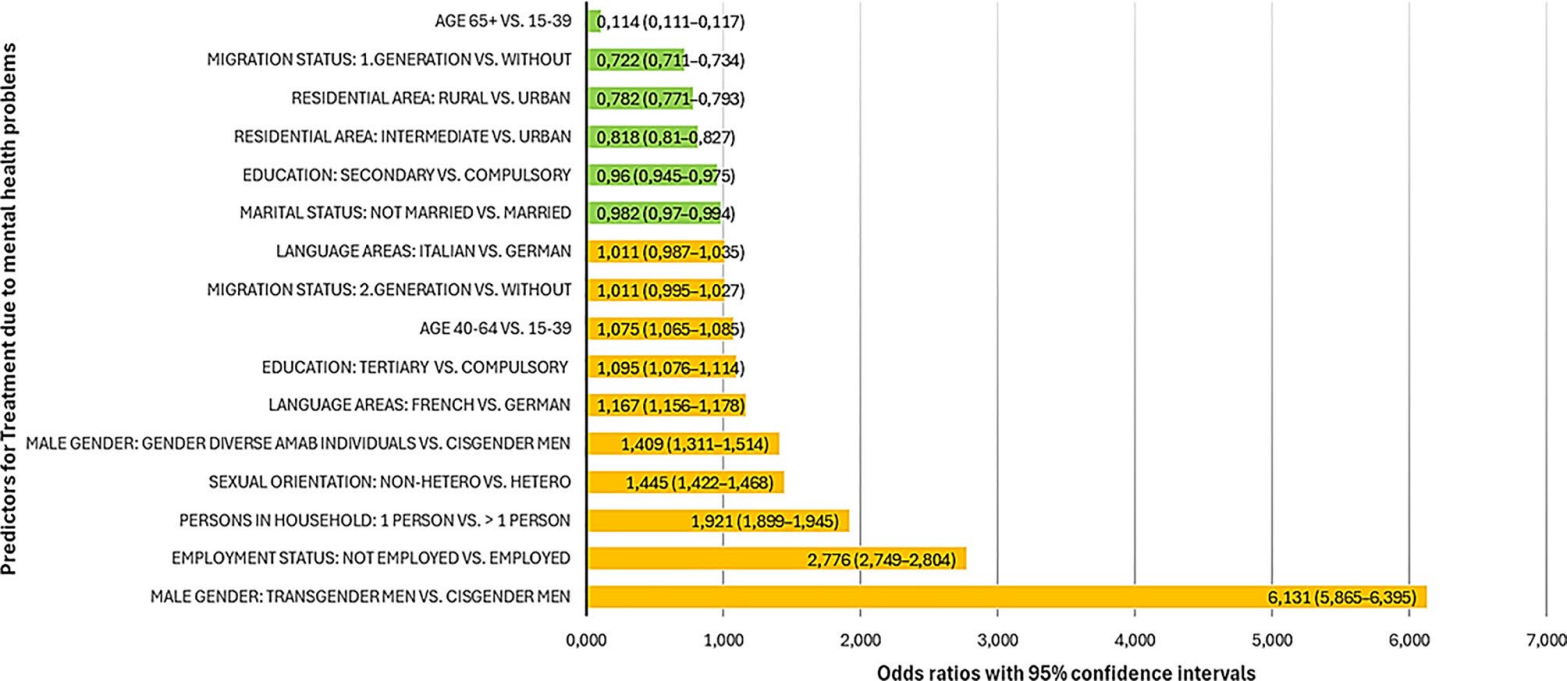
Odds ratios of predictors for treatment due to mental health problems

The binary logistic regression model predicting the likelihood of having utilized any treatment service due to a mental health problem in the 12 past months was statistically significant, χ² (16) = 96,113.76, *p* < .001, with a Nagelkerke R² of 0.078 and can be viewed in the supplementary Table 3. All predictors were statistically significant (*p* < .001) except for migration status (second-generation) and language area (Italian-speaking). The most prominent factors for having utilized a treatment service for mental health problems were being a transgender man (OR = 6.131), being unemployed (OR = 2.776), living in a single household (OR = 1.921), having a non-heterosexual orientation (OR = 1.445), and being a gender diverse AMAB individual (OR = 1.409). Other risk factors were living in a French-speaking region (OR = 1.167), tertiary education (OR = 1.095) and being middle aged (40–64, OR = 1.075). Factors associated with lower odds of utilizing mental health treatment were higher age (65+, OR=0.114), migration status (first-generation, OR = 0.722) and living in a rural (OR = 0.782) and intermediate (OR=0.818) area. Secondary education had an OR of 0.960 and not being married an OR of 0.982. All predictors and odds ratios are graphically displayed in Fig. 3.

#### Utilization of complementary medicine

**Fig. 4:**
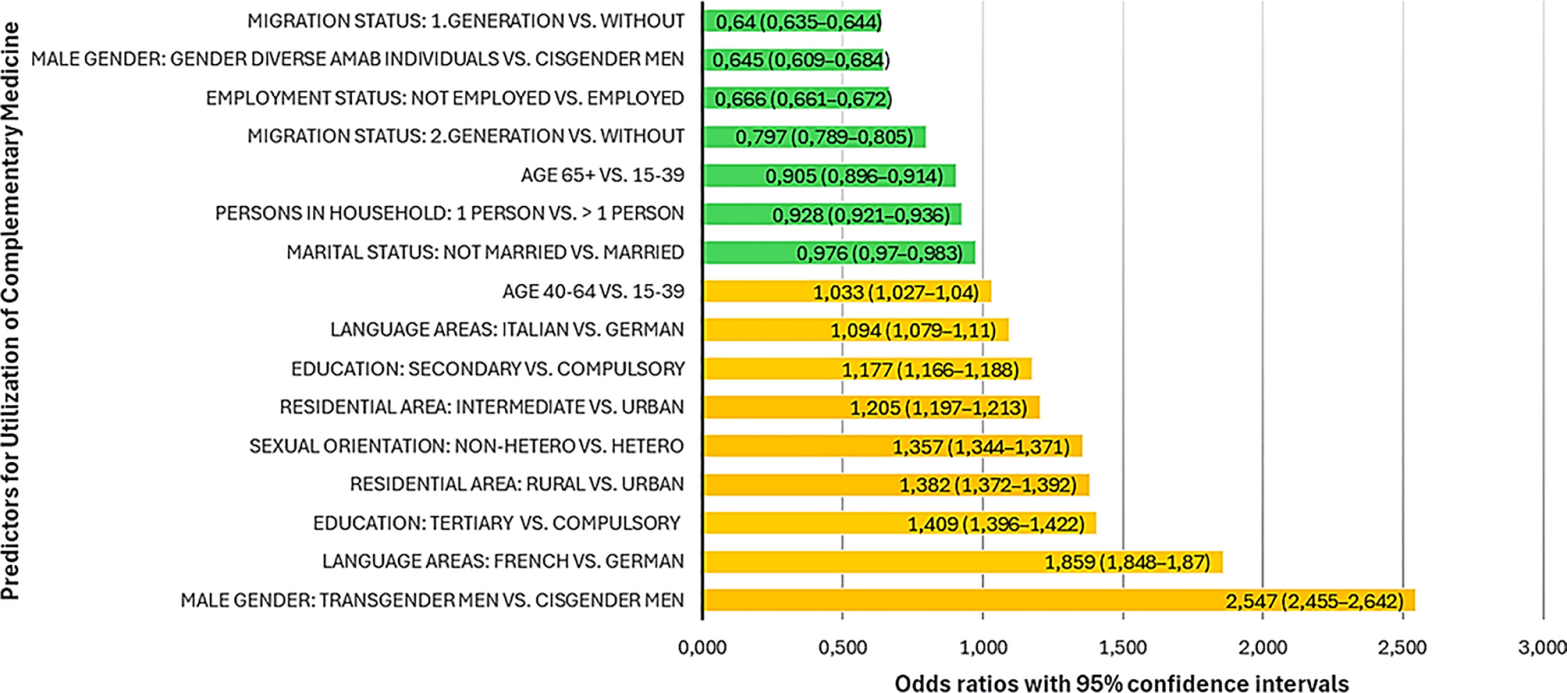
Odds ratios of predictors for utilization of complementary medicine

The binary logistic regression model predicting the likelihood of having utilized complementary medicine in the past 12 months was statistically significant, χ² (16) = 3,447,935.62, *p* < .001, with a Nagelkerke R² of 0.047. All predictors were statistically significant (*p* < .001). Details can be found in supplementary Table 4. The strongest factors associated with utilization of complementary medicine were being a transgender man (OR = 2.547), living in the French-speaking area of Switzerland (OR = 1.859), having a tertiary school education (OR = 1.409), living in a rural area (OR = 1.382), having a non-heterosexual orientation (OR = 1.357), living in an intermediate area (OR = 1.205) and secondary education (OR = 1.177). Other risk factors were living in an Italian-speaking region (OR = 1.094) and being middle aged (40–64, OR = 1.033). Factors associated with lower odds of utilizing complementary medicine were having a migration background (first-generation, OR=0.640, second-generation OR=0.797) and being a gender diverse AMAB individual (OR=0.645) and being unemployed (OR=0.666). Further protective factors were being aged 65+ (OR=0.905), living in a one-person household (OR=0.928) and not being married (OR=0.976). All predictors and odds ratios are graphically displayed in Fig. 4.

#### Robustness check controlling for outcome variables (SP and GP visits)

We first examined the correlation of all outcome variables (see supplementary Table 5). Since complementary medicine only had very weak correlations with all other forms of health care utilization, we excluded it from further analysis. We also excluded treatment due to mental health problems, since this might include treatments by a GP or SP and therefore overlap with our outcome variables. Including it as a predictor could introduce overlap and bias the results. For GP visits, results were nearly identical when SP visits were included, showing comparable effect sizes (IRR) and directions of associations for all variables (see supplementary Table 6). The most notable differences occurred for transgender men, where the IRR decreased from 1.113 to 1.050, and for unemployed men, where the IRR decreased from 1.303 to 1.215, indicating a small potential overlap in utilization patterns between SP and GP visits in these groups. Generally, SP visits were associated with 4.6% more GP visits. Although changes in specific variables were minor, including the number of GP visits as a predictor significantly improved model fit. This is shown by lower deviance (1,415,494 vs. 1,491,282), reduced information criteria (AIC: 10,457,299 vs. 10,554,881; BIC: 10,457,528 vs. 10,555,098), and higher log-likelihood (-5,228,632 vs. -5,277,424). While the Pearson chi-square ratio per degree of freedom was closer to 1 without GP visits (0.827 vs. 0.719), overall evidence supports including GP visits for enhanced explanatory power.

For SP visits, several differences in IRR were observed when GP visits were included in the model (see supplementary Table 7). The most notable changes occurred for transgender men, where the IRR dropped from 1.738 to 1.302, and for unemployed men, where it decreased from 1.870 to 1.613. Among men aged 65 + and those aged 40–64, the IRR declined from 1.020 to 0.766 and from 1.314 to 1.088, respectively. Other noteworthy shifts included tertiary education (increasing from 1.223 to 1.402), being gender diverse AMAB individual (dropping from 0.843 to 0.704), and men living in single-person households (decreasing from 1.303 to 1.175). For other variables, including migration background, the differences in IRR were only marginal. Rural areas became statistically significant (*p* < .001), although the IRR changed only slightly from 1.000 to 0.991. Overall, GP visits were associated with 10.6% more SP visits. The model including GP visits showed a significantly better fit, indicated by lower deviance (1.384 vs. 1.582), Pearson chi-square ratio (3.366 vs. 4.219), and information criteria (AIC: 9,255,355 vs. 11,046,585; BIC: 9,255,584 vs. 11,046,806). These results suggest that including GP visits improves predictive accuracy.

#### Robustness check controlling for supplemental health insurance for complementary medicine

When including the availability of supplemental health insurance in our model of complementary medicine utilization, several effects could be observed (see supplementary Table 8). Generally, having supplemental health insurance was associated with 3.044 higher odds of utilizing complementary medicine. Regarding other variables, the most notable difference occurred for transgender men, where the odds ratio dropped from 2.547 to 0.754, indicating a partial mediator effect of having a supplemental health insurance. For gender diverse AMAB individuals, the odds only slightly changed from 0.645 to 0.860. For men aged 65+, odds decreased from 0.905 to 0.797 and for non-heterosexual men odds dropped from 1.357 to 1.191, when accounting for supplemental health insurance For men aged 40–64 (1.033 → 0.950), those living in the Italian-speaking region (1.094 → 0.925), marital status (0.976 → 1.093), and living alone (0.928 → 1.023), odds were reversed, though the magnitude of these changes was marginal. For all other factors, including migration background, differences in odds were only modest and did not reverse direction. Including supplemental health insurance as a predictor substantially improved model fit. This is reflected in a lower − 2 Log-Likelihood (2,819,967.65 vs. 3,447,935.62) and higher explained variance (Cox & Snell R²: 0.075 vs. 0.031; Nagelkerke R²: 0.111 vs. 0.047). Overall, the increase in explained variance indicates that accounting for supplemental health insurance notably enhances the model’s explanatory power.

## Discussion

In this study, we used data derived from the SHS 2022 to examine healthcare utilization across diverse male gender subgroups as well as groups of different migration backgrounds, while accounting for key sociodemographic factors in Switzerland.

### Healthcare utilization in male gender subgroups

Univariate analyses showed that transgender men reported overall higher healthcare utilization compared to cisgender men, although effect sizes were small. The effect remained significant even after controlling for key sociodemographic factors, with transgender men exhibiting higher rates of healthcare utilization in terms of the number of GP and SP visits, as well as higher odds of mental health treatment. Our analyses further showed that transgender men rated the quality of perceived care similarly to cisgender men. Although the results were statistically significant, effect sizes were small to negligible, indicating no meaningful difference in perceived quality of care between these groups. Initially, these findings appear to contradict international studies suggesting that transgender individuals often show reduced healthcare utilization due to fears of potential discrimination and poor treatment [23, 26, 27, 54]. However, a previous Swiss study found more GP and psychologist visits among transgender and nonbinary individuals, although only the association with psychologist visits remained significant after adjusting for sociodemographic variables [29]. The higher healthcare utilization, we found in our study, may reflect greater health needs among transgender men, including poorer mental [32] and physical health [22, 30], as well as requirements related to gender-affirming care involving psychological and psychiatric consultations [29]. In Switzerland, accessing affirmative care typically requires a diagnostic assessment and may involve psychotherapy or psychiatric evaluation, with health insurance covering medically indicated treatments. However, the association between higher use and greater need is hypothetical and needs further investigation. Moreover, perceived quality of care was similar between transgender and cisgender men, suggesting that despite higher utilization, transgender men do not report worse experiences with the care they receive. However, higher utilization does not necessarily imply better access or overall quality of care. One additional possible explanation for the higher use of health services among transgender men is that, unlike cisgender men— who generally seek care less frequently than women [9–12] —transgender men may not yet have adopted these patterns of lower healthcare engagement. At the same time, it is also possible that transgender men delay seeking care due to potential barriers and experiences of discrimination. As a result, their healthcare needs may be higher by the time they finally do access services—contributing to the observed higher rate in utilization. When additionally controlling for SP and GP visits in the respective models, we found a notable decrease in utilization among transgender men, suggesting that part of their higher SP visit rate is linked to GP visits. This indicates overlap or influence between the two, likely because GPs often refer patients to SPs or because their health needs involve both providers, as is common in gender-affirming care. However, due to the cross-sectional study design, causal conclusions cannot be drawn, underscoring the necessity of future research.

When examining the use of complementary medicine, transgender men initially showed higher odds of utilization compared to cisgender men (OR = 2.547). However, after accounting for the availability of supplemental health insurance for complementary treatments, the association was reversed and markedly decreased (OR = 0.754), suggesting that insurance coverage partially mediates the observed difference in complementary medicine use. This change in odds after accounting for supplemental insurance may reflect the lower coverage of such insurance among transgender men (35.6% vs. 55.7% in cisgender men) in our study, which in turn may influence the likelihood of utilizing complementary medicine. This finding highlights the importance of considering structural and financial access factors in future research when interpreting gender differences in complementary medicine use.

For gender diverse AMAB individuals, a different pattern emerged. They also showed higher odds of mental health treatment, while reporting fewer GP and SP visits as well as lower use of complementary medicine compared to cisgender men. Adjusting for supplemental insurance slightly attenuated this association (OR increased from 0.645 to 0.860) but did not change the overall pattern of lower utilization. Similarly, the odds did not meaningfully change when controlling for GP and SP visits in the respective models, suggesting that there may be less substantial overlap or mediation between GP and SP visits in this group. The observed higher rate of mental health treatment may similarly be explained by poorer mental health [55, 56] and requirements for gender-affirming care [29], although this remains hypothetical and needs to be investigated further. The lower rate of utilization of other health care services, on the other hand, may reflect different barriers to accessing healthcare services or disparities in health care-seeking behavior in this group. One possible explanation could be dissatisfaction with perceived quality of care. In our sample, gender diverse AMAB individuals more often rated the quality of GP and SP visits as moderate and bad compared to cisgender and transgender men. An Austrian study likewise suggested that nonbinary individuals reported a weaker patient–physician relationship compared to transgender men [57]. Similarly, nonbinary individuals were less likely to feel comfortable during physical examinations and less likely to feel respected by healthcare providers compared to cisgender and transgender people [58]. It has been hypothesized that nonbinary individuals, whose identities differ more substantially from the binary notion of sex (men/women), are at a higher risk of discrimination [57]. However, more research is needed to better understand these disparities and their underlying mechanisms.

### Healthcare utilization by migration background

In univariate analyses, differences in healthcare utilization as well as perceived quality of care by migration background appeared significant, though effect sizes were minimal. In the corresponding regression models, men with a first-generation migration background showed slightly higher rates of frequent GP visits, but lower rates of SP visits. Additionally, they had lower odds of utilizing mental health care and complementary medicine. These findings partially align with previous research, suggesting that individuals with first-generation migration background are generally less likely to access medical care compared to those without a migration background [39]. A German review [38] similarly reported that individuals with migration background used SP services, psychosocial counselling and complementary medicine less frequently. Patterns of GP visits usually are more inconsistent, with some studies reporting no differences and others indicating higher use among individuals with first-generation migration background [38, 59]. Although the IRR of 1.06 indicates only a modest 6% higher level of GP visits in our study, this suggests that men with a first-generation migration background may concentrate their healthcare utilization in primary care – possibly due to barriers in accessing other services, such as language difficulties and limited health system knowledge [60]. This utilization pattern is supported by our statistical models, which additionally controlled for SP and GP visits and showed no substantial change in the IRR, suggesting that utilization of these services is only weakly related among this group and that more GP visits do not necessarily lead to more SP visits.

Men with a second-generation migration background showed no significant differences in SP visits or mental health care utilization. For GP visits, the difference compared to those without a migration background was only marginally significant, with an IRR of 1.006, indicating no meaningful effect. However, in terms of complementary medicine, men with a second-generation background had lower odds of utilization compared to those without a migration background. Previous research from Switzerland supports these findings, suggesting that people with a second-generation migration background often show no substantial differences in health care utilization compared to those without a migration background [39]. However, in terms of complementary medicine, individuals with a second-generation migration background seem to align more closely with those of the first-generation, as both groups showed lower utilization compared to individuals without a migration background. The German review mentioned above [38], reported generally lower use of complementary medicine among people with migration background. However, the studies included did not differentiate between people of first- and second-generation migration background. The lower utilization we observed among men with a second-generation migration background likely reflects a combination of structural and contextual factors, including language-related challenges, limited availability, personal preferences or health-system routines that may not fully accommodate diverse patient expectations. Importantly, this finding should not be interpreted as indicating poorer health outcomes in case of non-use, as the evidence base for many complementary medicine treatment is limited [17–20]. Rather, these patterns highlight differences in health service use across populations.

### Healthcare utilization by sociodemographic factors

We further examined key sociodemographic factors commonly associated with healthcare utilization, such as employment, age, education, marital status, household structure, sexual orientation, residential environment and language region and observed notable differences.

We found that unemployed men showed a higher rate of GP and SP visits, as well as higher odds of utilizing mental health care. However, use of complementary medicine was lower compared to employed men. Unemployment is often associated with poorer physical and mental health outcomes [61], which might lead to higher health care utilization. In the case of complementary medicine, utilization remained low, even after accounting for supplemental health insurance, possibly due to higher cost burdens, such as extra insurance fees or self-payments. Future research is necessary to further investigate this phenomenon and to explore potential causal associations. It is important to mention that our unemployed group both included non-employable men, such as pensioners, housemen, men in training as well as those that were unemployed but available for the job market within two weeks. A more differentiated analysis between these subgroups could lead to a more detailed picture.

With increasing age, we observed a higher rate of both GP and SP visits among older men (40–64 and 65+) compared to younger men (15–39). However, for men aged 65+, the higher rate in SP visits was only marginal (with an IRR of 1.020) and even decreased notably when controlling for GP visits (IRR=0.766). This indicates that the initially comparable rate of SP visits among older men (age 65+) may largely be explained by their higher frequency of GP visits. Once GP visits are considered, older men actually have fewer SP visits than younger men. The relatively higher rate of GP visits compared to SP visits observed in our study may reflect the tendency of older individuals (65+) to consult their GP more frequently than any other healthcare provider [62], possibly due to greater trust in GPs, reduced mobility or long waiting times for SP appointments. The higher GP use aligns with previous findings suggesting that health care utilization generally rises with age [63] due to a higher prevalence of chronic diseases and multimorbidity, which require more frequent medical consultations and treatments [64, 65]. Treatment of mental health problems, however, was associated with substantially lower odds among men aged 65 + compared to younger men (15–39), while there was a minimal elevation for those aged 40–64 compared to younger men (15–39). This pattern is consistent with previous research, which found that older individuals tend to have the lowest utilization rate for psychotherapeutic and psychiatric services, while middle-aged men show the highest use [63, 66]. A similar pattern was observed for complementary medicine. However, when adjusting for supplemental health insurance, men aged 65 + showed even lower odds (OR decreased from 0.905 to 0.797), while those aged 40–64 also had slightly lower odds (OR decreased from 1.033 to 0.950). This indicates that differences in complementary medicine use are partly mediated by insurance coverage in these groups. In addition, lower utilization may also reflect less openness to alternative treatment methods, a preference for more conventional approaches among older men, and potential financial considerations, including out-of-pocket costs or the cost of supplemental insurance itself.

For education, we found higher educational attainment (secondary and tertiary school education) to be associated with a lower rate of GP visits, but with a higher rate of SP visits compared to those with compulsory school education, which aligns well with previous research [63]. Higher education is often associated with higher health literacy [67], facilitating better recognition of health needs and therefore more targeted use of specialized health services. In our data, this was reflected by men with tertiary education showing higher SP utilization than those with compulsory education, with the IRR increasing from 1.223 to 1.402 after controlling for GP visits. This may indicate that the association between higher education and greater SP use persists, and may even be more pronounced, after accounting for GP visit frequency. Tertiary education was also associated with higher odds of mental health service utilization, aligning with findings from another Swiss study [66]. This finding may also be explained by higher health literacy [67] and therefore greater awareness of available services among individuals with higher education. Complementary medicine use was similarly higher among men with higher education, and controlling for supplemental health insurance had little impact on this association. The higher utilization of complementary medicine among men with higher education may reflect personal preferences, greater interest in alternative health options, or higher awareness of available services, although further research is necessary to explore these hypotheses.

We did not find relevant differences in marital status, except for a slightly higher use of SP visits among married men, which has previously been attributed to greater social support and encouragement from spouses to seek specialized healthcare [68].

Men living in a one-person household reported higher use of GP, SP and mental health care compared to those living with others. This may result from reduced informal support at home, increasing reliance on formal care. Previous research reports that persons living alone had a higher likelihood of being hospitalized and utilizing emergency departments and GP services [41, 42]. While men living alone initially appeared to use complementary medicine slightly less frequently (OR = 0.928), adjusting for supplemental health insurance reversed this association (OR = 1.023), indicating that differences in insurance coverage may partially explain the observed pattern. However, the effects in both directions were relatively small.

Another important sociodemographic factor was sexual orientation. Non-heterosexual men reported a higher number of GP visits, a higher likelihood of receiving mental health treatment, and a greater use of complementary medicine compared to heterosexual men. In contrast, SP visits were slightly lower among non-heterosexual men. Adjusting for supplemental health insurance reduced the odds of complementary medicine use from 1.357 to 1.191, although they remained higher than for heterosexual men. According to the Minority Stress Model (MSM), sexual minorities frequently experience worse mental and physical health outcomes due to discrimination stressors [35, 69], suggesting a higher need for health care [70]. Previous research confirmed that sexual minorities are often faced with an increased need for healthcare, but also highlighted constraints in access to care, due to discrimination [71]. The slightly higher number of GP visits reported by non-heterosexual men in our study may be understood in light of findings by Agénor et al. [72], who observed that sexual minority men tend to cultivate close relationships with their primary care providers through the intentional selection of “safe providers” and the need for ongoing, continuous care. The lower use of specialist services, on the other hand, could result from hesitation to seek specialized care due to barriers, such as fears of discrimination or concerns about being misunderstood [73].

Regarding residential areas, GP visits differed only minimally between rural, intermediate and urban regions, indicating nearly equivalent utilization across these areas. SP visits were lower in intermediate areas, which may reflect limited specialist availability or access outside urban centers [74], although utilization in rural areas did not differ compared to urban areas. It has been hypothesized that residents from rural areas may be more accustomed to traveling longer distances for specialist care [74], resulting in similar utilization to urban areas. However, more research is necessary to examine this association. We further found lower mental health treatment in rural or intermediate areas compared to urban areas, consistent with previous research [66]. This may be attributed to limited availability of services in these regions [66] or a lower prevalence of mental health problems [75]. The relatively higher use of complementary medicine in rural and intermediate areas compared to urban ones in our study might be explained by differences in healthcare-seeking behaviors or availability of conventional services, although more research seems necessary to understand these patterns.

We further found that men residing in French- and Italian-speaking parts reported fewer GP visits than those in German-speaking regions. For SP visits, it was less for Italian-, but more for French-speaking parts. Regarding mental health utilization, there was no difference for men in Italian-speaking parts, while men in the French-speaking region showed higher utilization than those in the German-speaking region. Use of complementary medicine was higher in both the Italian- and French-speaking parts; however, the difference in the Italian-speaking region was only minimal and reversed when accounting for supplemental health insurance. Overall, health care utilization varies across linguistic regions in Switzerland, potentially reflecting systemic factors that may influence health behaviors and access to care. Differences may partly reflect service availability, as greater access often leads to higher utilization [76]. Additionally, a study [77] found higher health literacy in German-speaking regions, along with stronger personal interest in health matters, which may explain the higher GP visit rates there. The higher use of complementary medicine in French- and Italian-speaking regions could reflect either greater availability of such services or distinct local preferences for alternative approaches. Further research is needed to better understand regional differences in health care utilization.

### Limitations and future directions

Several limitations should be noted. The cross-sectional design and observational nature of our study prevents any conclusions about causality. Any explanations or mechanisms discussed in the main text should be regarded as tentative hypotheses rather than established causal relationships. Overall, our findings primarily highlight patterns of healthcare utilization rather than their underlying causes. Further, the use of self-reported data may introduce biases, such as social desirability, which could have influenced participants’ reports of healthcare utilization. For instance, participants may have overreported visits to appear responsible about their health, or underreported mental health service use due to perceived stigma—i.e., negative social attitudes or personal discomfort associated with seeking mental health care—potentially affecting observed group differences. In addition, the dataset lacks information on medical and social transition status among transgender individuals and gender diverse AMAB individuals, as well as the country of origin for those with a migration background – both of which could influence healthcare utilization.

The two-step approach to categorize gender variables enabled the identification of transgender and gender-diverse respondents. However, the limited response options may not fully capture the diversity of gender identities in Switzerland. Moreover, the small number of respondents identifying as non-binary, other, or “I don’t know” required us to combine these respondents into a single gender-diverse AMAB category. Although this grouping helps prevent misclassification, it results in a heterogeneous category and limits analytic precision. Additionally, the small, unweighted sample sizes in some subgroups (e.g. gender diverse AMAB individuals: *N* = 16) limited statistical power and call for cautious interpretation of these results. They also restricted our ability to examine potential interaction effects and to conduct a full intersectional analysis. Future population-based surveys could address these measurement constraints by expanding gender identity response options, oversampling gender minority groups, or using adaptive sampling strategies, which have been shown to improve the accuracy and inclusiveness of gender measurement [45, 78].

Another limitation of our study is the relatively high proportion of missing values for GP visits (24.7% in the weighted and 22.6% in the unweighted sample), which resulted in the exclusion of a substantial number of observations from the respective regression analyses. This may introduce selection bias, thereby affecting the representativeness of the analytical samples and the generalizability of our findings. A plausible explanation for the discrepancy in missing data regarding visits to GPs vs. SPs could be that participants found it harder to recall routine GP contacts than the typically rarer and more salient SP visits, leading to more item nonresponse for GP visits.

Finally, general health care insurance type (basic mandatory or supplementary/private) was not collected while household income could not be included due to substantial missing data; both may impact healthcare access and utilization and should be considered in future studies.

Despite these limitations, the study’s large and representative sample constitutes a major strength, as it increases the generalizability of the results and provides meaningful insights into health care utilization among men in Switzerland. An additional strength lies in the distinction between different male gender identities, which enables a more detailed understanding of how these identities relate to health care use.

### Summary and conclusion

Overall, we found that different male gender identities and migration background differ regarding healthcare utilization in Switzerland. Transgender men showed higher use of healthcare services, which may reflect greater health needs, including gender-affirming care, although this association needs further investigation in future studies. Perceived care quality was similar between transgender and cisgender men, suggesting that despite higher healthcare utilization, transgender men do not report worse experiences. However, higher utilization does not necessarily indicate better access or overall quality of care. It remains unclear whether this higher utilization is also a consequence of delayed care-seeking due to barriers or fear of discrimination, which may contribute to greater health needs and higher utilization rates. In contrast, gender diverse AMAB individuals exhibited lower use of GP and SP care but higher mental health service use, which may be explained by potential barriers and dissatisfaction with care. These findings underscore the need for more inclusive, identity-sensitive healthcare and further research on gender-diverse populations.

Our findings further indicate that first-generation migrant men in Switzerland tend to use primary care services (GPs) slightly more but access SPs, mental health care and complementary medicine less frequently than men without migration background. Men with a second-generation migration background showed health care utilization patterns largely similar to men without a migration background, except for lower use of complementary medicine. These differences likely reflect a combination of structural and contextual factors—such as language barriers, limited service availability, personal preferences and organizational practices within the health system that may not fully accommodate diverse patient needs. These findings highlight the need to reduce system-level barriers to promote equitable access for all migrant groups and should be further investigated in future studies.

## Supplementary Information

Below is the link to the electronic supplementary material.

Supplementary Material 1

Supplementary Material 2

## Data Availability

The data used in this study are owned by the Swiss Federal Statistical Office (SFSO) and cannot be shared publicly due to licensing restrictions. However, the data can be requested directly from the SFSO upon completion of a data-use contract. Access may be subject to a fee depending on the scope of the request.

## Abbreviations

SHS: Swiss Health Survey
GP: General Practitioner
SP: Specialist Physician
MHP: Mental health problems
IRR: Incidence Rate Ratio
OR: Odds Ratio
SD: Standard Deviation
SE: Standard Error
B: Regression Coefficient
