# Supplementary material for: Determinants of male healthcare utilization in Switzerland: does gender identity and migration background matter?
Source: BMC Health Serv Res. 2026 Feb 27;26:336. doi: 10.1186/s12913-026-14185-y (PMC12967004; doi:10.1186/s12913-026-14185-y)
Supplement: Supplementary file 1 — Supplementary Material 1 [file 12913_2026_14185_MOESM1_ESM.docx]

Supplementary File 1: Assessment of health care utilization

1. General Practitioner:
   1. In the past 12 months, how many times have you visited a general practitioner or family doctor?
   2. How would you rate the overall quality of your last visit to a general practitioner or family doctor?

Excellent 🞎 very good 🞎 good 🞎 moderate 🞎 bad 🞎

1. Specialist Physician:
   1. In the past 12 months, how many times have you visited a specialist physician?
   2. How would you rate the overall quality of your last visit to a specialist physician?

Excellent 🞎 very good 🞎 good 🞎 moderate 🞎 bad 🞎

1. Treatment due to mental health problem:
   1. In the past 12 months, have you received treatment for a mental health problem?

Yes 🞎 No🞎

- 1. Which type(s) of service(s) did you use for mental health treatment?

🞎 Psychologist/Psychotherapist

🞎 Psychiatrist

🞎 General Practitioner

🞎 Other Physician

🞎 Complementary and alternative medicine practitioner

🞎 Other non-medical therapist

1. Complementary and Alternative Medicine:

In the past 12 months…

- 1. … how many sessions or treatments of complementary or alternative medicine have you received for health-related problems (such as acupuncture, homeopathy, shiatsu, or classical massage)?
  2. … how many times have you visited an osteopath?
  3. … how many times have you visited an alternative practitioner?
  4. Do you have supplementary insurance for complementary medicine?

Yes 🞎 No🞎 I don’t know 🞎

Note: The questionnaire only represents an excerpt from the Swiss Health Survey 2022. It was administerd in German, French and Italian and has not undergone a formal translation procedure.
