# Supplementary material for: Determinants of male healthcare utilization in Switzerland: does gender identity and migration background matter?
Source: BMC Health Serv Res. 2026 Feb 27;26:336. doi: 10.1186/s12913-026-14185-y (PMC12967004; doi:10.1186/s12913-026-14185-y)
Supplement: Supplementary file 2 — Supplementary Material 2 [file 12913_2026_14185_MOESM2_ESM.docx]

Supplementary Table 1. Negative Binomial Regression Predicting the Number of GP Visits in the Past 12 Months

| **Predictor** | **B** | **SE** | **Wald χ²** | **IRR (Exp[B])** | **95% CI for IRR** | **Sig.** |
| --- | --- | --- | --- | --- | --- | --- |
| Intercept | .955 | .003 | 94531.96 | 2.599 | [2.583, 2.614] | p<.001 |
| **Male Gender** |  |  |  |  |  |  |
| Cisgender Men | Ref. |  |  |  |  |  |
| Transgender men | .107 | .011 | 93.922 | 1.113 | [1.089, 1.137] | p<.001 |
| Gender Diverse AMAB individuals | -,123 | .017 | 50.926 | .884 | [.855, .914] | p<,001 |
| **Migration Background** |  |  |  |  |  |  |
| Without | Ref. |  |  |  |  |  |
| Migration: 1st gen vs. none | .058 | .002 | 1033.452 | 1.060 | [1.056, 1.064] | p<.001 |
| Migration: 2nd+ gen vs. none | .006 | .003 | 4.182 | 1.006 | [1.000, 1.012] | p=.041 |
| **Age** |  |  |  |  |  |  |
| Age: 15-39 | Ref. |  |  |  |  |  |
| Age: 40–64 | .226 | .002 | 13539.695 | 1.254 | [1.249, 1.258] | p<.001 |
| Age: 65+ | .141 | .003 | 2663.778 | 1.151 | [1.145, 1.158] | p<.001 |
| **Language Area** |  |  |  |  |  |  |
| German-speaking | Ref. |  |  |  |  |  |
| French-speaking | -.103 | .002 | 3215.287 | .902 | [.899, .906] | p<.001 |
| Italian-speaking | -.069 | .004 | 332.200 | .933 | [.926, .941] | p<.001 |
| **Residential Area** |  |  |  |  |  |  |
| Urban | Ref. |  |  |  |  |  |
| Intermediate | .026 | .002 | 201.720 | 1.026 | [1.022, 1.030] | p<.001 |
| Rural | .017 | .002 | 64.775 | 1.017 | [1.013, 1.021] | p<.001 |
| **Education** |  |  |  |  |  |  |
| Compulsory | Ref. |  |  |  |  |  |
| Secondary | -.142 | .003 | 3213.614 | .868 | [.863, .873] | p<.001 |
| Tertiary | -.321 | .003 | 15875.294 | .726 | [.721, .730] | p<.001 |
| **Marital Status** |  |  |  |  |  |  |
| Married | Ref. |  |  |  |  |  |
| Not married | .003 | .002 | 2.622 | 1.003 | [.999, 1.007] | p=.105 |
| **Persons in household** |  |  |  |  |  |  |
| > One-person | Ref. |  |  |  |  |  |
| One-person | .037 | .002 | 283.432 | 1.038 | [1.034, 1.042] | p<.001 |
| **Sexual Orientation** |  |  |  |  |  |  |
| Heterosexual | Ref. |  |  |  |  |  |
| Non-heterosexual | .078 | .003 | 691.084 | 1.081 | [1.075, 1.087] | p<.001 |
| **Employment** |  |  |  |  |  |  |
| Employed | Ref. |  |  |  |  |  |
| Not employed | .265 | .002 | 16491.516 | 1.303 | [1.298, 1.309] | p<.001 |

Note: Analytical Sample Size: N= 6,284; Weighted cases: N=2,466,676. Excluded cases: N=2,415 (27,8%, unweighted), N=1,039,125 (29.7%, weighted), AMAB: assigned male at birth

Supplementary Table 2. Negative Binomial Regression Predicting the Number of SP Visits in the Past 12 Months

| **Predictor** | **B** | **SE** | **Wald χ²** | **IRR (Exp[B])** | **95% CI for IRR** | **Sig.** |
| --- | --- | --- | --- | --- | --- | --- |
| Intercept | -.110 | .003 | 1323.937 | .896 | [.891, .901] | p<.001 |
| **Male Gender** |  |  |  |  |  |  |
| Cisgender Men | Ref. |  |  |  |  |  |
| Transgender men | .553 | .011 | 2584.780 | 1.738 | [1.701; 1.776] | p<.001 |
| Gender Diverse AMAB individuals | -.171 | .017 | 103.324 | .843 | [.815; .871] | p<.001 |
| **Migration Background** |  |  |  |  |  |  |
| Without | Ref. |  |  |  |  |  |
| Migration: 1st gen vs. none | -.085 | .002 | 2552.391 | .919 | [.915; .922] | p<.001 |
| Migration: 2nd+ gen vs. none | -.001 | .003 | .046 | .999 | [.991; 1.005] | p=.831 |
| **Age** |  |  |  |  |  |  |
| Age: 15-39 | Ref. |  |  |  |  |  |
| Age: 40–64 | .273 | .002 | 21900.658 | 1.314 | [1.309; 1.319] | p<.001 |
| Age: 65+ | .020 | .003 | 58.902 | 1.020 | [1.014; 1.026] | p<.001 |
| **Language Area** |  |  |  |  |  |  |
| German-speaking | Ref. |  |  |  |  |  |
| French-speaking | .230 | .002 | 18758.078 | 1.259 | [1.254; 1.264] | p<.001 |
| Italian-speaking | -.167 | .004 | 1913.239 | .846 | [.840; .853] | p<.001 |
| **Residential Area** |  |  |  |  |  |  |
| Urban | Ref. |  |  |  |  |  |
| Intermediate | -.120 | .002 | 4387.212 | .887 | [.883; .890] | p<.001 |
| Rural | .000 | .002 | .017 | 1.000 | [.996; 1.004] | p=.895 |
| **Education** |  |  |  |  |  |  |
| Compulsory | Ref. |  |  |  |  |  |
| Secondary | .050 | .003 | 412.829 | 1.051 | [1.045; 1.057] | p<.001 |
| Tertiary | .201 | .003 | 6402.749 | 1.223 | [1.215; 1.230] | p<.001 |
| **Marital Status** |  |  |  |  |  |  |
| Married | Ref. |  |  |  |  |  |
| Not married | .113 | .002 | 3756.681 | 1.120 | [1.115; 1.124] | p<.001 |
| **Persons in household** |  |  |  |  |  |  |
| > One-person | Ref. |  |  |  |  |  |
| One-person | 0.265 | .002 | 17362.938 | 1.303 | [1.298; 1.309] | p<.001 |
| **Sexual Orientation** |  |  |  |  |  |  |
| Heterosexual | Ref. |  |  |  |  |  |
| Non-heterosexual | -.015 | .003 | 26.289 | .985 | [.979; .991] | p<.001 |
| **Employment** |  |  |  |  |  |  |
| Employed | Ref. |  |  |  |  |  |
| Not employed | .626 | .002 | 105414.435 | 1.870 | [1.863; 1.877] | p<.001 |

Note: Analytical Sample Size: N= 8,121; Weighted cases: N=3,276,871. Excluded cases: N=578 (6.6%, unweighted), N=228,930 (6.5%, weighted). AMAB: assigned male at birth.

Supplementary Table 3. Binary Logistic Regression Predicting Treatment due to mental health problems

| **Predictor** | **B** | **SE** | **Wald** | **OR**  **(Exp [B])** | **95% CI for OR** | **Sig.** |
| --- | --- | --- | --- | --- | --- | --- |
| **Male Gender** |  |  |  |  |  |  |
| Cisgender Men | Ref. |  |  |  |  |  |
| Transgender men | 1.812 | .022 | 7067.88 | 6.131 | [5.865;6.395] | p<.001 |
| Gender Diverse AMAB individuals | .343 | .039 | 76.78 | 1.409 | [1.311;1.514] 1.1.52] | p<.001 |
| **Migration Background** |  |  |  |  |  |  |
| Without | Ref. |  |  |  |  |  |
| Migration: 1st gen vs. none | -.326 | .006 | 3238.526 | .722 | [.711;.734] | p<.001 |
| Migration: 2nd+ gen vs. none | .011 | .008 | 2.135 | 1.011 | [.995;1.027] | p=.144 |
| **Age** |  |  |  |  |  |  |
| Age: 15-39 | Ref. |  |  |  |  |  |
| Age: 40–64 | .072 | .005 | 173.02 | 1.075 | [1.065;1.085] | p<.001 |
| Age: 65+ | -2.171 | .012 | 35377.22 | .114 | [.111;.117] | p<.001 |
| **Language Area** |  |  |  |  |  |  |
| German-speaking | Ref. |  |  |  |  |  |
| French-speaking | .154 | .005 | 813.58 | 1.167 | [1.156;1.178] | p<.001 |
| Italian-speaking | .011 | .012 | .899 | 1.011 | [.987; 1.035] | p=.343 |
| **Residential Area** |  |  |  |  |  |  |
| Urban | Ref. |  |  |  |  |  |
| Intermediate | -.201 | .006 | 1086.11 | .818 | [.810;.827] | p<.001 |
| Rural | -.246 | .007 | 1166.01 | .782 | [.771;.793] | p<.001 |
| **Education** |  |  |  |  |  |  |
| Compulsory | Ref. |  |  |  |  |  |
| Secondary | -.041 | .008 | 28.25 | .960 | [.945;.975] | p<.001 |
| Tertiary | .091 | .008 | 137.28 | 1.095 | [1.076;1.114] | p<.001 |
| **Marital Status** |  |  |  |  |  |  |
| Married | Ref. |  |  |  |  |  |
| Not married | -.018 | .006 | 9.13 | .982 | [.970;.994] | p<.001 |
| **Persons in household** |  |  |  |  |  |  |
| > One-person | Ref. |  |  |  |  |  |
| One-person | .653 | .006 | 11896.75 | 1.921 | [1.899;1.945] | p<.001 |
| **Sexual Orientation** |  |  |  |  |  |  |
| Heterosexual | Ref. |  |  |  |  |  |
| Non-heterosexual | .368 | .008 | 2218.53 | 1.445 | [1.422;1.468] | p<.001 |
| **Employment** |  |  |  |  |  |  |
| Employed | Ref. |  |  |  |  |  |
| Not employed | 1.021 | .006 | 32049.85 | 2.776 | [2.749;2.804] | p<.001 |

Note: The dependent variable was coded as follows: no=0, yes=1. Analytical sample: N=8,141; Weighted cases: 3,284,833.Excluded cases: N=558 (6.4%, unweighted), N=220,968 (6.3% weighted). AMAB: assigned male at birth.

Supplementary Table 4. Binary Logistic Regression Predicting the Utilization of Complementary Medicine

| **Predictor** | **B** | **SE** | **Wald** | **OR**  **(Exp [B])** | **95% CI for OR** | **Sig.** |
| --- | --- | --- | --- | --- | --- | --- |
| **Male Gender** |  |  |  |  |  |  |
| Cisgender Men | Ref. |  | 2731.022 |  |  |  |
| Transgender men | .935 | .019 | 2501.141 | 2.547 | [2.455; 2.642] | <.001 |
| Gender Diverse AMAB individuals | -.438 | .029 | 222.091 | .645 | [.609; .684] | <.001 |
| **Migration Background** |  |  |  |  |  |  |
| Without | Ref. |  | 18819.525 |  |  |  |
| Migration: 1st gen vs. none | -.447 | .003 | 18430.896 | .640 | [.635; .644] | <.001 |
| Migration: 2nd+ gen vs. none | -.227 | .005 | 2101.270 | .797 | [.789; .805] | <.001 |
| **Age** |  |  |  |  |  |  |
| Age: 15-39 | Ref. |  | 806.247 |  |  |  |
| Age: 40–64 | .033 | .003 | 100.220 | 1.033 | [1.027; 1.040] | <.001 |
| Age: 65+ | -.100 | .005 | 381.476 | .905 | [.896; .914] | <.001 |
| **Language Area** |  |  |  |  |  |  |
| German-speaking | Ref. |  | 42412.620 |  |  |  |
| French-speaking | .620 | .003 | 42171.271 | 1.859 | [1.848; 1.870] | <.001 |
| Italian-speaking | .090 | .007 | 155.918 | 1.094 | [1.079; 1.110] | <.001 |
| **Residential Area** |  |  |  |  |  |  |
| Urban | Ref. |  | 9019.616 |  |  |  |
| Intermediate | .186 | .003 | 3217.629 | 1.205 | [1.197; 1.213] | <.001 |
| Rural | .323 | .004 | 7938.315 | 1.382 | [1.372; 1.392] | <.001 |
| **Education** |  |  |  |  |  |  |
| Compulsory | Ref. |  | 6949.431 |  |  |  |
| Secondary | .163 | .005 | 1132.699 | 1.177 | [1.166; 1.188] | <.001 |
| Tertiary | .343 | .005 | 5033.754 | 1.409 | [1.396; 1.422] | <.001 |
| **Marital Status** |  |  |  |  |  |  |
| Married | Ref. |  |  |  |  |  |
| Not married | -.024 | .003 | 51.395 | .976 | [.970; .983] | <.001 |
| **Persons in household** |  |  |  |  |  |  |
| > One-person | Ref. |  |  |  |  |  |
| One-person | -.074 | .004 | 360.360 | .928 | [.921; .936] | <.001 |
| **Sexual Orientation** |  |  |  |  |  |  |
| Heterosexual | Ref. |  |  |  |  |  |
| Non-heterosexual | .305 | .005 | 3657.252 | 1.357 | [1.344; 1.371] | <.001 |
| **Employment** |  |  |  |  |  |  |
| Employed | Ref. |  |  |  |  |  |
| Not employed | -.406 | .004 | 10130.606 | .666 | [.661; .672] | <.001 |

Note: The dependent variable was coded as follows: no=0, yes =1. Analytical sample: N=8,113; Weighted cases:

3,277,097. Excluded cases: N=586 (6.7%, unweighted), N=228,704 (6.5%, weighted). AMAB: assigned male at

birth

Supplementary Table 5. Spearman Correlation Matrix for Health Care Utilization*

| Variable | GP visits | SP visits | Treatment due to mental problem | Complementary medicine |
| --- | --- | --- | --- | --- |
| GP visits | -- | .227^a^ | .150^b^ | .053^c^ |
| SP visits | .227^a^ | -- | .170^d^ | .083^e^ |
| Treatment due to mental problem | .150^b^ | .170^d^ | -- | .067^f^ |
| Complementary medicine | .053^c^ | .083^e^ | .067^f^ | -- |

Note: *All correlations were significant on a p<.001 level. GP: General Practitioner, SP: Specialist Physician. Analytical Sample Size: a. unweighted: N=6,719, weighted cases: N=2,632,977; excluded cases: N=1,980 (22.8%, unweighted), N=872,824 (24.9%, weighted); b. unweighted: N=6,729, weighted cases: N=2,637,429; excluded cases: N=1,970 (22.6%, unweighted), N=868,372 (24.8%, weighted); c. unweighted: N=6,694, weighted cases: N=2,624,792; excluded cases: N=2,005 (23.1%, unweighted), N=881,009 (25.1%, weighted);
d. unweighted: N=8,669, weighted cases: N=3,493,294; excluded cases: N=30 (0.3%, unweighted), N=12,507 (0.4%, weighted); e. unweighted: N=8,629, weighted cases: N=3,479,050; excluded cases: N=70 (0.8%, unweighted), N=26,751 (0.8%, weighted); f. unweighted: N=8,649, weighted cases: N=3,487,236; excluded cases: N=50 (0.6%, unweighted), N=18,565 (0.5%, weighted).

Supplementary Table 6. Negative Binomial Regression Predicting the Number of GP Visits in the Past 12 Months: Robustness Analysis Including SP Visits

| **Predictor** | **B** | **SE** | **Wald χ²** | **IRR (Exp[B])** | **95% CI for IRR** | **Sig.** |
| --- | --- | --- | --- | --- | --- | --- |
| Intercept | .906 | .003 | 83831,706 | 2.474 | [2.460;2.489] | **<.001** |
| **Male Gender** |  |  |  |  |  |  |
| Cisgender Men | Ref. |  |  |  |  |  |
| Transgender men | .049 | .011 | 19.823 | 1.050 | [1.027;1.074] | <.001 |
| Gender Diverse AMAB individuals inindividuals identities | -.088 | .017 | 26.031 | .916 | [.885;.947] | <.001 |
| **Migration Background** |  |  |  |  |  |  |
| Without | Ref. |  |  |  |  |  |
| Migration: 1st gen vs. none | .057 | .002 | 991.587 | 1.059 | [1.054;1.062] | <.001 |
| Migration: 2nd+ gen vs. none | .006 | .003 | 4.711 | 1.006 | [1.001;1.012] | .030 |
| **Age** |  |  |  |  |  |  |
| Age: 15-39 | Ref. |  |  |  |  |  |
| Age: 40–64 | .192 | .002 | 9694.282 | 1.212 | [1.207;1.217] | <.001 |
| Age: 65+ | .179 | .003 | 4228.547 | 1.196 | [1.189;1.202] | <.001 |
| **Language Area** |  |  |  |  |  |  |
| German-speaking | Ref. |  |  |  |  |  |
| French-speaking | -.150 | .002 | 6706.599 | .861 | [.857;.863] | <.001 |
| Italian-speaking | -.046 | .004 | 148.114 | .955 | [.947;.962] | <.001 |
| **Residential Area** |  |  |  |  |  |  |
| Urban | Ref. |  |  |  |  |  |
| Intermediate | .043 | .002 | 529.932 | 1.044 | [1.040;1.048] | <.001 |
| Rural | -.005 | .002 | 4.448 | .995 | [.991;1.000] | .035 |
| **Education** |  |  |  |  |  |  |
| Compulsory | Ref. |  |  |  |  |  |
| Secondary | -.165 | .003 | 4306.686 | .848 | [.844;0.852] | <.001 |
| Tertiary | -.342 | .003 | 17882.353 | .710 | [.707;.714] | <.001 |
| **Marital Status** |  |  |  |  |  |  |
| Married | Ref. |  |  |  |  |  |
| Not married | .003 | .002 | 1074.339  1,958 | 1.003 | [.999;1.007] | .162 |
| **Persons in household** |  |  |  |  |  |  |
| > One-person | Ref. |  |  |  |  |  |
| One-person | -.019 | .002 | 75.354 | .981 | [.976;.985] | <.001 |
| **Sexual Orientation** |  |  |  |  |  |  |
| Heterosexual | Ref. |  |  |  |  |  |
| Non-heterosexual | .097 | .003 | 1074.339 | 1.102 | [1.095;1.108] | <.001 |
| **Employment** |  |  |  |  |  |  |
| Employed | Ref. |  |  |  |  |  |
| Not employed | .195 | .002 | 8693.708 | 1.215 | [1.210;1.221] | <.001 |
| **SP visits** | .045 | .000 | 56652.859 | 1.046 | [1.046;1.046] | <.001 |

Note: Analytical sample: N=6,270; Weighted cases: 2,460,591. Excluded cases: N=2,429 (27.9%, unweighted), N=1,045,210 (29.8%, weighted). AMAB: assigned male at birth.

Supplementary Table 7. Negative Binomial Regression Predicting the Number of SP Visits in the Past 12 Months: Robustness Analysis Including GP Visits

| **Predictor** | **B** | **SE** | **Wald χ²** | **IRR (Exp[B])** | **95% CI for IRR** | **Sig.** |
| --- | --- | --- | --- | --- | --- | --- |
| Intercept | -.026 | .004 | 57.488 | 0.974 | [.968;.981] | <0.001 |
| **Male Gender** |  |  |  |  |  |  |
| Cisgender Men | Ref. |  |  |  |  |  |
| Transgender men | .264 | .012 | 521.093 | 1.302 | [1.273;1.332] | <0.001 |
| Gender Diverse AMAB individuals identities | -.351 | .018 | 389.389 | .704 | [.680;.728] | <0.001 |
| **Migration Background** |  |  |  |  |  |  |
| Without | Ref. |  |  |  |  |  |
| Migration: 1st gen vs. none | -.091 | .002 | 2303.714 | .913 | [.910;.917] | <0.001 |
| Migration: 2nd+ gen vs. none | .013 | .003 | 18.62 | 1.013 | [1.007;1.018] | <0.001 |
| **Age** |  |  |  |  |  |  |
| Age: 15-39 | Ref. |  |  |  |  |  |
| Age: 40–64 | .084 | .002 | 1605.614 | 1.088 | [1.083;1.092] | <0.001 |
| Age: 65+ | -.266 | .003 | 8916.393 | .766 | [.763;.771] | <0.001 |
| **Language Area** |  |  |  |  |  |  |
| German-speaking | Ref. |  |  |  |  |  |
| French-speaking | .249 | .002 | 17729.988 | 1.282 | [1.279;1.288] | <0.001 |
| Italian-speaking | -.151 | .004 | 1318.205 | .860 | [.853;.867] | <0.001 |
| **Residential Area** |  |  |  |  |  |  |
| Urban | Ref. |  |  |  |  |  |
| Intermediate | -.115 | .002 | 3359.478 | .891 | [.888;.895] | <0.001 |
| Rural | -.009 | .002 | 16.611 | .991 | [.986;.995] | <0.001 |
| **Education** |  |  |  |  |  |  |
| Compulsory | Ref. |  |  |  |  |  |
| Secondary | .087 | .003 | 1004.215 | 1.091 | [1.085;1.096] | <0.001 |
| Tertiary | .338 | .003 | 14650.803 | 1.402 | [1.395;1.410] | <0.001 |
| **Marital Status** |  |  |  |  |  |  |
| Married | Ref. |  |  |  |  |  |
| Not married | .109 | .002 | 2787.51 | 1.115 | [1.111;1.120] | <0.001 |
| **Persons in household** |  |  |  |  |  |  |
| > One-person | Ref. |  |  |  |  |  |
| One-person | .161 | .002 | 5182.199 | 1.175 | [1.170;1.179] | <0.001 |
| **Sexual Orientation** |  |  |  |  |  |  |
| Heterosexual | Ref. |  |  |  |  |  |
| Non-heterosexual | .011 | .003 | 12.386 | 1.011 | [1.005;1.017] | <0.001 |
| **Employment** |  |  |  |  |  |  |
| Employed | Ref. |  |  |  |  |  |
| Not employed | .478 | .002 | 50249.342 | 1.613 | [1.606;1.621] | <0.001 |
| **GP visits** | .101 | .0003 | 148066.297 | 1.107 | [1.106;1.1074] | <0.001 |

Note: Analytical sample: N=6,270; Weighted cases: 2,460,591. Excluded cases: N=2,429 (27.9%, unweighted), N=1,045,210 (29.8%, weighted). AMAB: assigned male at birth.

Supplementary Table 8. Binary Logistic Regression Predicting the Utilization of Complementary Medicine: Robustness Analysis including SHI for Complementary Medicine

| **Predictor** | **B** | **SE** | **Wald χ²** | **OR (Exp[B])** | **95% CI for OR** | **Sig.** |
| --- | --- | --- | --- | --- | --- | --- |
| Intercept | -2.079 | .007 | 82696.282 | .125 |  | <0.001 |
| **Male Gender** |  |  |  |  |  |  |
| Cisgender Men | Ref. |  |  |  |  |  |
| Transgender men | -.282 | .030 | 87.134 | .754 | .711;.800 | <0.001 |
| Gender Diverse AMAB individuals identities | -.150 | .032 | 21.959 | .860 | .808;.916 | <0.001 |
| **Migration Background** |  |  |  |  |  |  |
| Without | Ref. |  |  |  |  |  |
| Migration: 1st gen vs. none | -.296 | .004 | 6665.541 | .744 | .739;.749 | <0.001 |
| Migration: 2nd+ gen vs. none | -.200 | .006 | 1222.073 | .819 | .810;.828 | <0.001 |
| **Age** |  |  |  |  |  |  |
| Age: 15-39 | Ref. |  |  |  |  |  |
| Age: 40–64 | -.051 | .004 | 201.221 | .950 | .944;.957 | <0.001 |
| Age: 65+ | -.227 | .006 | 1641.557 | .797 | .788;.806 | <0.001 |
| **Language Area** |  |  |  |  |  |  |
| German-speaking | Ref. |  |  |  |  |  |
| French-speaking | .625 | .003 | 35227.547  99 | 1.868 | 1.855;1.880 | <0.001 |
| Italian-speaking | -.078 | .008 | 99.800 | .925 | .911;.939 | <0.001 |
| **Residential Area** |  |  |  |  |  |  |
| Urban | Ref. |  |  |  |  |  |
| Intermediate | .111 | .004 | 942.442 | 1.118 | 1.110;1.126 | <0.001 |
| Rural | .269 | .004 | 4492.942 | 1.309 | 1.299;1.319 | <0.001 |
| **Education** |  |  |  |  |  |  |
| Compulsory | Ref |  |  |  |  |  |
| Secondary | .198 | .006 | 1104.895 | 1.219 | 1.205;1.233 | <0.001 |
| Tertiary | .318 | .006 | 2907.986 | 1.374 | 1.358;1.390 | <0.001 |
| **Marital Status** |  |  |  |  |  |  |
| Married | Ref. |  |  |  |  |  |
| Not married | .089 | .004 | 597.184 | 1.093 | 1.085;1.101 | <0.001 |
| **Persons in household** |  |  |  |  |  |  |
| > One-person | Ref. |  |  |  |  |  |
| One-person | .023 | .004 | 29.356 | 1.023 | 1.015;1.032 | <0.001 |
| **Sexual Orientation** |  |  |  |  |  |  |
| Heterosexual | Ref. |  |  |  |  |  |
| Non-heterosexual | .175 | .006 | 980.697 | 1.191 | 1.178;1:205 | <0.001 |
| **Employment** |  |  |  |  |  |  |
| Employed | Ref. |  |  |  |  |  |
| Not employed | -.350 | .005 | 6006.819 | .705 | .698;.711 | <0.001 |
| **SHI for complementary medicine**  No  Yes | Ref.  1.113 | .003 | 118972.709 | 3.044 | 3.025;3.064 | <0.001 |

Note: Analytical sample: N=6,823; Weighted cases: 2,688,154. Excluded cases: N=1,876 (21.6%, unweighted), N=817,647 (23.3%, weighted). SHI: supplemental health insurance; AMAB: assigned male at birth
